# Comprehensive review on rhino-neurosurgery

**DOI:** 10.3205/cto000116

**Published:** 2015-12-22

**Authors:** Werner Hosemann, Henry W.S. Schroeder

**Affiliations:** 1 Department of Otolaryngology, Head & Neck Surgery, University Medicine Greifswald, Germany; 2 Department of Neurosurgery, University Medicine Greifswald, Germany

**Keywords:** skull base surgery, rhino-neurosurgery, endoscopic endonasal sinus surgery

## Abstract

In the past 2 decades, an innovative and active field of surgical collaboration has been evolved and established combining the expertise of neurosurgery and rhinosurgery in the endonasal treatment of different lesions affecting the anterior skull base together with the adjacent intranasal and intradural areas. Important prerequisites for this development were improvements of technical devices, definitions of transnasal surgical corridors, and approvements in endonasal reconstructions, e.g. by use of pedicled nasal mucosal flaps. Due to these improvements, the rate of perioperative infectious complications remained acceptable. Interdisciplinary surgical teams (4-hands-2-minds) have been established constituting specialized centers of “rhino-neurosurgery”. With growing expertise of these groups, it could be shown that oncological results and perioperative complications were comparable to traditional surgery while at the same time the patients’ morbidity could be reduced.

The present review encompasses the recent literature focusing on the development, technical details, results, and complications of “rhino-neurosurgery”.

## Abbreviations

A1 – A1 segment of the anterior cerebral artery

A2 – A2 segment of the anterior cerebral artery

Acom – Anterior communicating artery

BA – Basilar artery

C – Optic chiasm

CC – Cavernous section of the internal carotid artery

CL – Clivus

CLC – Clival segment of the internal carotid artery

CSF – Cerebrospinal fluid

F – Fornix

ICA – Internal carotid artery

MI – Massa intermedia

NF – Nasoseptal flap

O – Optic nerve

OC – Oculomotor nerve

PC – Choroid plexus

PCA – Posterior cerebral artery

PCL – Posterior clinoid process

PG – Pituitary gland

PL – Planum sphenoidale

PS – Pituitary stalk

S – Sella

SCA – Superior cerebellar artery

T – Tumor

Tri – Trigeminal nerve

Tro – Trochlear nerve

TS – Tuberculum sellae

VN – Nerve of pterygoid canal (“Vidian nerve”)

White star – Lateral optico-carotid recess

White dot – Medial optico-carotid recess

## 1 Introduction

During the last 3 decades, endoscopic endonasal sinus surgery has been established as a standard procedure in the field of ENT-specific surgical interventions for chronic inflammatory diseases of the nasal mucosa. The surgical techniques have become more and more elaborate and differentiated and the indication spectrum has been enlarged also to ENT specific endonasal skull base surgery. With this development, the nasal cavity and the paranasal sinuses were no longer simply locations of local foci of disease, alternatively they became part of the natural corridor of advanced surgical interventions. Several extradural interventions were defined in the area of the paranasal neighboring structures, if needed they were completed by more or less circumscribed (<2 cm) resections of the dura with the according reconstruction. Examples are surgeries of angiofibromas, inverted papillomas, circumscribed meningo-encephaloceles, or selected less advanced malignomas (“MIER = **M**inimally **I**nvasive **E**ndoscopic **R**esection of sinonasal and skull base malignant neoplasms”; less appropriate: “ECFR = **E**ndoscopic **C**ranio**F**acial **R**esection”) [1], [2], [3], [4], [5], [6], [7], [8], [9], [10], [11] (Table 1 (Tab. 1)).

In the interval, the routine use of endoscopes and angular optics was established in neurosurgery, first as further development of the already very early introduced ventricular neuroendoscopy and as individually used technical completion in cases of traditional microscopic microsurgical interventions. Subsequently, the concept encompassed other target structures in the sense of (exclusive) endoscopic neurosurgery or endoscope-assisted microneurosurgery (combined use of microscope and endoscope), which led to different types of transcranial skull base endoscopy and to merely endoscopic intraventricular interventions as for example ventriculostomy (“ETV = **E**ndoscopic **T**hird **V**entriculostomy”), aqueductoplasty with stenting, tumor resections, removal of colloid cysts, and fenestration of arachnoid, pineal, or intraparenchymal cysts.

Regarding the area of the anterior skull base, the development led step-by-step to endonasal, exclusively endoscopic pituitary surgery, without creating a sublabial or transseptal access and without applying special specula (e.g. as “FEPS = **F**unctional **E**ndoscopic **P**ituitary **S**urgery”). Endonasal interventions can be considered as variation of endoscopically controlled microneurosurgery or “**N**atural **O**rifice **T**ransluminal **E**ndoscopic **S**urgery (NOTES)” [10], [12], [13], [14], [15], [16], [17], [18], [19], [20], [21], [22], [23], [24], [25], [26], [27], [28], [29], [30], [31], [32], [33], [34] (Table 1 (Tab. 1)).

Due to the continuous improvement of endoscopes, camera systems (HDTV), monitors, and microsurgical instruments in combination with optimized imaging (e.g. CT scan, MRI) and the optimized perioperative concomitant therapy a special interdisciplinary endonasal surgery could develop, in particular in the overlapping disciplines of neurosurgery and ENT surgery (“EEA = **E**xpanded **E**ndonasal **A**pproach”, “EEEA = **E**xtended **E**ndoscopic **E**ndonasal **A**pproach”) [25], [35], [36], [37], [38], [39], [40]. The development was promoted by the fact that most lesions of the anterior skull base shift their neurovascular neighboring structures in lateral, rostral, or posterior directions and thus they can be reached relatively carefully and directly through a transnasal corridor [41]. Another milestone of the development was the definition of pedicled intranasal flaps for reconstruction. Its application reduced the rate of postoperative CSF fistulas to an acceptable level. The emerging activities were summarized with the term of “rhino-neurosurgery” (alternatively “endoneurosurgery”, “endoscopic neurosurgery”, or “neurorhinology”). The terms of “rhinoneurosurgery” and “simultaneous neuro-rhinosurgery” had already been used for interdisciplinary interventions at the skull base without limitation to a certain type of surgical approach and on the basis of mostly successive contribution of both involved disciplines [42], [43]. In the course of time, to express the described particular development of endoscopic endonasal surgery techniques, the term of “rhino-neurosurgery” has achieved its special meaning of interdisciplinary endonasal endoscopic skull base surgery, including the direct cooperation of neurosurgeons and ENT surgeons (“4 hands 2 minds”; “2 nostrils, 4 hands”) [37], [38].

The present review describes the basic principles, the current spectrum, and the special risks of this rhino-neurosurgical field of activity. The focus will be placed on practical aspects of the cooperation of ENT and neurosurgeons in cases of interdisciplinary endonasal or transnasal interventions at the anterior skull base, the sphenoid sinus and the sella, as well as the clivus, pterygoid process, and the upper part of the cervical spine with the respective, specific work load at the dura and in the adjacent endocranium. This review does not compete with established surgery manuals, monographs, or other reviews [44], [45], [46], [47], [48], [49], [50], [51].

Regarding the general analysis of treatment results of single lesions with an extensive review of the literature until the year 2010, repeatedly the **“European Position Paper on Endoscopic Management of Tumors of the Nose, Paranasal Sinuses, and Skull Base”** is referred to [52].

Aspects of “neuroendoscopy” or intradisciplinary endoscopic neurosurgery (e.g. as regular pituitary gland surgery or in a sense of extranasal interventions with visualization of the ventricles or deep cerebral structures) are not emphasized [53]. Furthermore, with regard to endonasal interventions of the orbita or in relation to exclusive ENT-activities concerning expanded extradural endoscopic surgery, other articles and monographs must be mentioned. Regarding aspects of general endoscopic surgery techniques and their complications or e.g. the routinely performed closure of dura defects, other papers in this issue are mentioned [3], [11]. The current literature up to March 2014 was included in this paper. The abundance of the according literature is impressive, even if an apparent redundancy cannot be neglected.

## 2 Basic principles of rhino-neurosurgery

### 2.1 Surgical concepts, absolute and relative contraindications

Via nares and nasal cavities, rhino-neurosurgical interventions have the merit of using a natural, i.e. minimally invasive, access within the midface. In comparison to conventional neurosurgical surgeries, incisions of the outer skin and also other visible results of manipulations at the external skull bones can be avoided, not to mention side effects of the temporary intraoperative retraction of brain tissue for exposition of target structures. The aim is a reduced intra- and postoperative morbidity as well as a shorter reconvalescence of the patient [30], [36], [54].

If the indication is made correctly, transnasal surgeries lead directly to the epicenter of the pathology. Often a tumor of the anterior skull base compresses the surrounding neurovascular structures and thus impairs their function to a critical extent. Accessing the lesion via appropriate transnasal corridors, it is possible to reach the pathologic focus by careful preparation without increasing the risk of critically injuring neighboring neurovascular structures.

In the further course, the inhomogeneity of the surgical target structures allows two basic strategies:

**a.** In cases of smaller (pseudo-)tumors, the lesion is exposed with a primary dissection of the tumor periphery (“outside-in approach”).

**b.** More often the alternative sequence is performed with exposition of the lesion followed by internal debulking of the tumor and extracapsular dissection of the tumor margins after tumor collapse (“inside-out approach”). The early debulking allows primary decompression of sensitive neurovascular structures.

**Figure 1:**
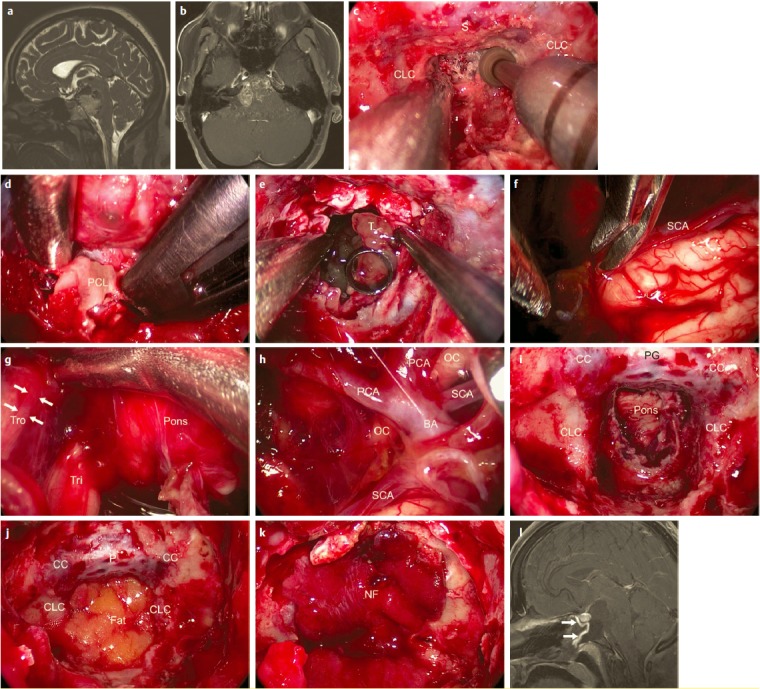
Example of endoscopic endonasal resection of a chordoma via a transclival approach. a, b The 36-year-old female patient presented with sudden headaches and paresis of the oculomotor nerve. Imaging revealed a large hemorrhagic, contrast enhancing petroclival tumor with extension in suprasellar direction and into the right cerebellopontine angle. The tumor led to a significant compression of the mesencephalon and pons. During preparation for surgery, the patient developed ophthalmoplegia on the right side and a high-grade hemiparesis on the left. Because of the progredient neurological deficits, surgery was performed as an emergency intervention. c Resection of the clivus with the high-speed drill with presentation of the clival internal carotid artery and the sellar dura. In order to create enough space for microsurgical preparation, the roof of the clival carotid artery was removed on both sides and the sella was completely decompressed. d Resection of the posterior clinoid process in order to reach cranial tumor parts. e Tumor resection with the curette. f Bimanual sharp separation of the tumor capsula from the superior cerebellar artery. g Inspection of the cerebellopontine angle with the 45° optic after removal of the tumor shows the trochlear and trigeminal nerves. h Inspection in cranial direction with the 45° optic reveals the basilar tip with the efferent arteries as well as the oculomotor nerve on both sides. i Presentation of the transclival approach which was used for resection of the large chordoma. j A fat graft is glued into the clival skull base defect. k Reconstruction of the skull base with a nasoseptal flap. l The postoperative MRI shows complete tumor resection and the nasoseptal flap well supplied with blood (arrows). The neurological deficits were completely regredient soon after surgery.

In both cases, the intervention is finalized with special reconstructive measures [2], [37], [38], [55], [56] (Figure 1 (Fig. 1)).

En bloc resection of the tumors is generally not the primary objective of rhino-neurosurgical interventions even if the procedure generally corresponds de facto to a local removal of pathological tissue equivalent to traditional transfacial or transcranial interventions [2], [30], [36], [54]. The main objective is a gross total resection (GTR) if it can be achieved without endangering the patient or risking functional deficiencies. If necessary (e.g. in case of meningiomas and chordomas) surrounding parts of the dura and the bone should be removed. A subtotal resection is often considered as acceptable if GTR cannot be achieved [57], [58].

To assess the result of complete resection, adequate imaging is performed for control examination. A “subtotal resection” is defined as incomplete tumor resection of more than 80% of the volume. “Partial tumor resection” corresponds to an excision of 50–80% of the tumor tissue, and in cases of less than 50% the tumor resection is designated “insufficient” [59], [60]. The last two categories are also summarized as “partial tumor resection” [61]. Alternative classifications mention other terms such as “subtotal resection” (or “near total resection”) in cases of more than 90% or even more than 95% of the tumor volume, whereas “partial resection” is achieved between 50% and 95% or below 50% [62], [63], [64], [65]. Other authors define “near total resection” when the residual tumor is less than 1 ml [66]. Rhino-neurosurgical interventions are naturally heterogeneous, corresponding to the diversity of possible lesions or diseases and the wide range of affected individual microanatomical structures with their texture and biomechanics.

Despite all this, rhino-neurosurgical interventions can be classified in a conceptual systematic. According to Schwartz et al. [30], [67], the difference is made between 4 different corridors in the nose and up to about 9 different approaches in the area of the skull base with specific anatomical target structures (Table 2 (Tab. 2)) [30]. The different corridors may be combined if needed [67].

Kassam et al. [37], [38], [49], [68] presented a similar, however, clearly more differentiated classification. It consists of a first rough classification of interventions in the sagittal level, i.e. at the median skull base. Laterally, this part is delineated by the internal carotid artery in the segment from the foramen lacerum to the anterior clinoid process including the cisternal segment. In anterior-posterior direction, this corridor reaches from the crista galli to the odontoid process. The sagittal level is opposed to “paramedian accesses” in the “coronary level”. Those are interventions with lateral orientation and target structures situated laterally to the mentioned parts of the carotid artery.

A detailed subclassification is done referring to the sagittal accesses with 7 modules and referring to the paramedian accesses with 9 modules, each with an exact definition of the corridor, the anatomical limits and the tangent CSF spaces or cisterns, neurovascular or anatomical key structures, and the most frequently encountered pathological processes [39], [55], [68], [69] (Table 3 (Tab. 3), Table 4 (Tab. 4)).

Further classifications were given by Castelnuovo and Locatelli [70]. Kasemsiri et al. [71] classified the transpterygoid approaches into 5 subtypes (see below). Kassam et al. [39] presented an alternative systematic of interventions in the paraclival space with orientation at the internal carotid artery.

The individual intervention is oriented at the described modules and corridors. It has to be modified and adapted according to the single case. The need for such modification is present for example in children [72]. In other cases, several transnasal interventions are combined or combinations of transnasal and transpalatine, transoral or transcranial accesses are performed in one or several sessions [73], [74]. En bloc resection is generally not the objective [75], [76] – however, in single cases it may become possible, e.g. in cases of reduced craniotomy combined with an endonasal intervention [77]. Systematic combinations of interventions performed in several sessions have been presented as “cranionasal approach” [78].

#### Absolute and relative contraindications

Often seen pathological entities subjected to rhino-neurosurgical treatment are summarized in Table 5 (Tab. 5).

Endonasal, rhino-neurosurgical interventions are generally not without alternative – hence relative and absolute indications have to be weighed out carefully [79], [80]. General limitations are:

- By individual calculation before a transnasal intervention, the chance of complete tumor resection with its risks must be considered as at least equivalent in comparison to conventional surgery from outside [81]. In cases of extended bilateral transnasal interventions at the anterior skull base, e.g. meningiomas, a loss of olfaction often cannot be avoided – which is not always the case for alternative transcranial interventions. The pros and cons have to be weighed out individually [82]. Endoscopic endonasal interventions can be combined sequentially or simultaneously with traditional interventions. Additionally, it must be noted that also the so-called traditional transcranial, transoral, or transfacial interventions have changed enormously and have been improved by the use of optic devices and after minimization of the accesses [83], [84], [85], [86], [87], [88], [89].
- Patients who have to undergo rhino-neurosurgical surgery must not have severe comorbidities that restrict longer interventions [10], [36], [38].
- If a crucial neurovascular structure is found ventral or medial to the target structure, i.e. in the corridor in front of the lesion, a transnasal approach is definitely not recommended. Subsequently, the most important rule of transnasal surgery is still: “never cross the nerves!” [36], [38], [39], [55].
- Rhino-neurosurgical interventions must only be performed with sufficient surgical expertise and appropriate technical equipment. The staff-related preconditions must be fulfilled for surgeries of long durations [36], [38], [39], [90].

In the literature the following contraindications are discussed as well:

- Extension of the (pseudo-) tumor clearly (more than 1 cm) beyond the lateral borders of direct exposition. Below the mentioned distance, the deficits of straight line of vision on the lesion may be compensated by angular optics and appropriate micro-instruments. On the other hand, for example in older patients, leaving of small remnants of slowly growing tumors may be strategically reasonable in individual cases [30], [91], [92], [93].
- Supraorbital extensions of malignomas, actually also the supraorbital extension of benign neoplasms, e.g. over the meridian of the orbita in lateral direction or from the sphenoid planum beyond the posterior roof of the ethmoid [75], [94], [95], [96].
- Tumors located laterally of the optic nerve, laterally of the distal internal carotid artery or the vertebral artery; tumors located laterally and posteriorly of the Gasserian ganglion; tumors in dorso-lateral direction of the inferior brain nerves, i.e. with relevant lateral processes at the foramen magnum in cases of transclival interventions [36], [55], [97], [98], [99], [100], [101], [102], [103], [104], [105], [106].
- More advanced tumor infiltration of the brain tissue; recurrences of malignomas near the brain with local scarring [2], [5], [90], [91], [94], [96], [107], [108], [109]; meningiomas with infiltration of the pia mater [110]. However, the involvement of the brain is not always considered as contraindication [55].
- Highly vascularized neoplasms [2]. Recurrences with fibrosis and masking or destruction of anatomical landmarks [111]. Generally, however, a condition after previous transcranial surgery is no contraindication [112]. On the other hand, other authors emphasize that vascularization, fibrosis, or calcification are no relevant aspects in the context of indication [39]. In single cases, even a palliative intervention may be indicated [113], [114].
- Involvement of relevant neurovascular structures in the tumor (e.g. encasement of the carotid artery, the anterior cerebral artery, the vertebral artery, or the optic nerve) or necessity of resection (with reconstruction, if required) of larger vessels. It is not recommended to routinely resect tumors of the cavernous sinus, in general also tumors with parasellar extension and an even only partly involvement of neurovascular structures [30], [36], [38], [39], [55], [94], [104], [109], [110], [112], [115], [116]. Extensions of tuberculum sellae meningiomas into the canal of the optic nerve are sometimes discussed critically [94]. Other authors, however, explicitly state that not every involvement of neurovascular structures is considered as absolute contraindication [93].
- Very large tumors are generally controlled more safely by conventional interventions. Depending on the pathological entity, a limit value is a tumor diameter of >4 cm or a volume of >40–80 cm³ [58], [76], [110], [117], [118], [119], [120]. The size of a tumor is considered as predictor of subtotal resection as well as of surgical complications [112]. Meningiomas of the tuberculum sellae are classified as critical with a tumor diameter of more than 2 or 3 cm in diameter [121], [122]. The same is true for large craniopharyngiomas with extension to the foramen of Monro [123], for clival chordomas of more than 20 cm³ [124] or for encephaloceles with external deformation of the face or originating from the posterior wall of the frontal sinus [125]. Other authors do not consider the tumor volume as being a limiting parameter of rhino-neurosurgical interventions [38], [39].
- In case of malignomas: Involvement of the orbital apex, invasion via the inferior orbital fissure or via the periorbita (indication for orbital exenteration). Involvement of the peripheral bony maxilla, the hard palate, or the peripheral frontal sinus, nasal bone, or the greater sphenoid wing. Infiltration of the optic nerve, the oropharynx, the lacrimal ducts, or the skin or facial soft parts [2], [5], [9], [36], [55], [75], [90], [91], [96], [105], [107], [112], [126].
- Intraorbital tumors of the superior and lateral quadrants (superior and lateral to the optic nerve in the orbita) [26], [30], [69], [127], [128].
- Transsphenoid interventions especially in children with a “conchal pneumatization” of the sphenoid sinus [109], [129]. Because of the generally reduced pneumatization, a short distance of the carotid arteries and generally narrow anatomical conditions, often no sufficiently wide transsphenoid corridor can be created in children below the age of 3–4 years. Children with small noses often require sublabial incision or an additional or alternative conventional surgical access. In general interventions in children with an assumed higher blood loss must be planned in several sessions [36], [72], [130], [131], [132], [133], [134], [135] – in single cases, however, appropriate endonasal interventions (e.g. meningoceles of the ethmoid plate) could be performed in children also for example in their first year of life [136], [137].
- Pathological processes below the “naso-axial”, “naso-palatine”, or “palatine” line (see below) or with extensions inferior to C2 [76], [104], [138], [139], [140], [141].
- An acute infection in the surgical corridor can require postponing or even interruption of the surgery with the possibility to continue after conservative therapy [36], [120].
- Tumors in cases of transpterygoid interventions, which grow around the cavernous, petrosal, or parapharyngeal carotid artery or which are located dorsal to the artery [142], [143]. It is further not recommended to perform interventions in which specific control of the vertical carotid artery in the most superior neck is needed or generally with significantly increased needs and difficulties of local hemostasis [144]. Generally it must be taken into account that endonasal manipulations during interventions on lateral parts of the infratemporal fossa are technically difficult. In such cases, the alternative or additional transoral access allows relatively short distances to reach the target structure with better instrumental ranges of action [145], [146], [147], [148], [149].
- Tumors of the infratemporal fossa with extended infiltration of the soft tissue, e.g. above the zygomatic arch [2], [116].
- Processes in the parapharyngeal space below the level of the nasal floor or with displacement of the big vessels in anterior direction [146].
- Relevant intracranial parts in case of a lesion, e.g. in the area of the infratemporal fossa or the foramen ovale [143], [150].

### 2.2 Microanatomical basics

A particular characteristic of the most recent development of rhino-neurosurgery is the significant intensification of scientific investigations on the micro-anatomy at the crossroads of the disciplines of neurosurgery and ENT surgery.

The neurosurgical anatomy with its “view from above” has already been described in detail in the literature [151], [152], [153], [154], [155]. The same is true for the known ENT specific literature on endonasal, endoscopic surgery of the paranasal sinuses and the anterior skull base.

In the last years, a multitude of detailed analyses were added with the special aspect of “endoscopic micro-anatomy”. The following general explanations may serve as introduction:

An ENT surgeon is very familiar with the basic anatomy of the **sphenoid sinus **as well as a neurosurgeon is familiar with the basic anatomy of the pituitary gland [154], [155], [156], [157]. The extent of pneumatization of the sphenoid sinus and the petrous bone correlate – in 40%, the lateral sphenoid sinus is in contact with the petrous apex and thus facilitates the creation of a surgical corridor [158]. In the sphenoid sinus, in nearly half of the cases only a single intersphenoid septum is found. In other cases, up to 5 bony septa are observed, mostly with contact to the parasellar or paraclival internal carotid artery on one side [159], [160]. The bone over the anterior prominence of the carotid artery lining the sphenoid sinus is mostly thinner than bone covering the sella. Depending on the pneumatization, the thickness of the bony posterior wall of the sphenoid sinus amounts to 0.2–10 mm. Attention must be paid to the abundant venous plexus and the cisterns [161].

The upper third of the **clivus** reaches from the dorsum sellae to the level of the trigeminal nerve or the level of the entrance of the abducens nerve into the dura. To the level of the neural roots of the glossopharyngeal nerve and to the lower edge of the petroclival fissure, the middle third of the clivus is located. The lower border of the middle third corresponds in anterior direction to a bony prominence (“pharyngeal tubercle”) as adhesion point of the pharyngeal raphe (superior pharyngeal constrictor muscle). The inferior third ends at the foramen magnum [106], [162], [163]. In infero-lateral direction, a bony prominence is found, the jugular tubercle, which is located above the hypoglossal canal and medial to the foramen jugulare and postero-medial to the foramen lacerum [164]. The length of the clivus amounts to about 45 mm, the width to 11–14 mm [165]. Between both carotid arteries, the distance amounts to around 18 mm in the petro-clival segment [166]. The distance between the posterior clinoid process and the porus of the abducens nerve amounts to about 13 (12–17) mm [162]. The access to the foramen magnum can be enlarged by removing the anterior third of the occipital condyle without affecting the hypoglossal canal [167].

Beside the **anterior and posterior clinoid process**, at the transition of the intracavernous and paraclinoid carotid artery, a **medial clinoid process** can be defined in about 50%. This corresponds to the medial opticocarotid recess from an endonasal aspect [168]. In its maximal variation (4% of all cases), this process is developed as a complete bony ring around the carotid artery; it may make it difficult to access the parasellar region [169].

The extent of the anterior-posterior directed pneumatization of a **sphenoid sinus** was traditionally, i.e. for the purposes of pituitary surgery, described as “conchal” (prevalence max. 2%), “presellar” (2–20%), or “sellar” (50–90%). For the detailed planning of surgical corridors in the context of rhino-neurosurgery, the sellar type can be subclassified into 6 subtypes with reference to the posterior wall of the sella, the clivus, the nerve of the pterygoid canal (Vidian nerve), the sphenoid crista, and the VR line (imagined line between the foramen rotundum and the foramen canalis pterygoidi in the frontal plane) [170], [171]. The septation of the sphenoid sinus runs nearly in median-sagittal direction in only 2/3 of the cases; very often, a more or less complete septum affects the protrusion of the carotid artery. In cases of pituitary tumors, a stable intersphenoid septum seems to contribute to a growth of the tumor in the suprasellar direction [160], [172]. On the other hand, a pituitary adenoma often leads to the prominence of the sellar floor into the sphenoid sinus [173].

**Figure 2:**
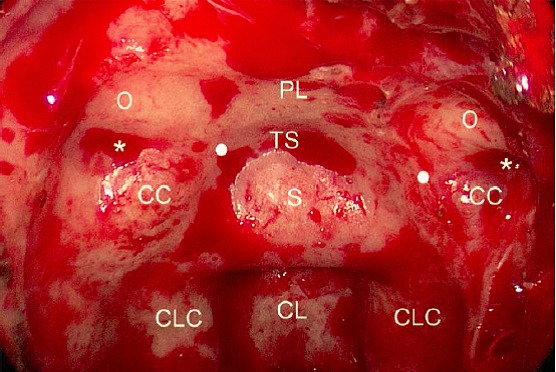
Posterior wall of the sphenoid sinus with the typical anatomical landmarks

The **lateral opticocarotid recess** (LOCR) in the sphenoid sinus corresponds to a pneumatization of the **optic strut**, an individually shaped bony strut between the underside of the anterior clinoid process and the sphenoid body (Figure 2 (Fig. 2)). It separates the optic nerve (located superior) from the medial superior orbital fissure (located inferior). In single cases, the further cranially located anterior clinoid process can be more or less completely pneumatized via the recess [171], [174], [175], [176], [177], [178], [179]. Below the superior orbital fissure, there is another, about 4 mm thick bony strut (**“maxillary strut”**) that separates the superior orbital fissure (cranial) from the foramen rotundum (caudal) [180], [181], [182]. It may serve as landmark for the dura exposition of the middle cranial fossa [182]. In the transition of sella, tuberculum sellae, carotid artery, optic nerve, and sphenoid planum, a more or less noticeable sinking of the bone is described in about 3/4 of the cases as **“medial optico-carotid recess”** (mOCR) which can be a landmark for broad exposition of parasellar and suprasellar structures. The peaked extension of this drop-shaped surface lies medially to the lateral opticocarotid recess that is the point where the carotid artery is located in the cavernous section of the optic nerve [176], [177], [180], [181], [183]. The **falciform ligament** corresponds to fold-like dura reinforcement that reaches from the anterior clinoid process above the optic nerve via the sphenoid planum in medial direction – this ligament often marks the anterior end of the dura opening in transplanum-transtuberculum interventions [176], [184], [185].

In 2 thirds of the cases, the sphenoid sinus also extends dorsal to a frontal plane through the posterior wall of the pituitary fossa (“clival recess”) [162]. Superiorly and anteriorly of the sella, the **tuberculum sellae** is located. From the view of the surgeon in cases of transcranial interventions, it corresponds to a small protrusion just above the sella in dorsal direction, below the pre-chiasmatic sulcus with the sphenoid limbus. In case of transnasal endoscopic interventions, no convex “tuberculum” is seen, at best a concavity that is consequently described as “suprasellar notch” or as “tubercular recess” [161], [178], [186], [187]. The optic chiasm is not located directly in the pre-chiasmatic sulcus, in the horizontal level it has a variable relation to the sella (in 15% each, a “pre-fixed” or “post-fixed” chiasm is found in front of or behind the diaphragm). Even the position of pituitary stalk in relation to the midpoint of the sella and also its angle with the vertical axis can vary [154], [187], [188], [189]. The angle, which is formed in the median-sagittal plane between the tuberculum septi and the descending part of the dorsal sphenoid planum, allows an anticipation of the range of exposition of the surgical site in cases of “transtubercular” interventions [186].

**Figure 3:**
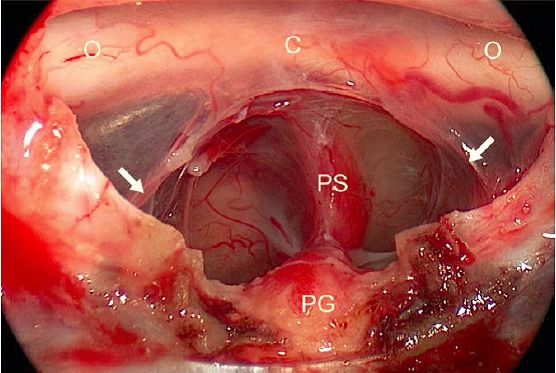
Endoscopic view with 30° optic on the suprasellar neuro-vascular structures (arrow: tractus hypophyseus superior)

The **sellar and pre-sellar structures** are very important for many rhino-neurosurgical interventions [190], [191]. The distance between both **internal carotid arteries** is a relevant parameter of the transsphenoid/transclival corridor. In the majority of the cases it is smallest in the area of the tuberculum (about 13 mm), amounts to about 16 mm at the level of the floor of the sphenoid sinus, and it is relatively high in the paraclival part (about 19 mm) – in single cases, however, severe individual variations (e.g. with an intercarotid distance in the area of the sella of only 4 mm or even “kissing carotid arteries”) can be observed [191], [192], [193], [194]. The width of the sella between the prominences of the A. carotis amounts to 13–22 mm [159], [195]. Those data correspond to an individually variable but mostly intraindividually bilateral equal course of the artery that is subdivided into 5–7 sections based on the generally anatomy and surgical view (Table 6 (Tab. 6)). From the point of view of transsphenoid endoscopic surgery, in the dorsolateral wall of the sphenoid sinus is a paraclival and in distal direction the parasellar segment of the artery (**paraclival and parasellar protuberance**). According to the general classification, the 2 larger segments correspond to 3 parts of the artery: part in the area of the foramen lacerum, cavernous, and clinoid part. The first 2 parts merge at the petrolingual ligament, the clinoid part is located between 2 defined spots of dura reinforcement (proximal and distal “ring”) [155], [184], [193], [196], [197], [198], [199], [200], [201], [202]. In the context of interventions, the petrous part of the artery is ‘protected’ from anterior by the mandibular nerve (N. V_3_) and by the tube [203]. In half of the cases, the carotid artery is located in the cavernous part directly at the pituitary gland [204]. From this part of the carotid artery, also the **inferior pituitary artery** originates reaching the gland on the infero-lateral side. The **superior pituitary artery** mostly departs from the intracranial part of the carotid artery and reaches mostly the upper third of the pituitary stalk, less frequently the middle or inferior third [12], [185] (Figure 3 (Fig. 3)). From a functional point of view, the superior pituitary artery is mostly essential, but not the inferior one [205]. Within the cavernous sinus, 3–4 branches of the carotid artery depart to the cranial nerves and in direction of the tentorium [206].

**Figure 4:**
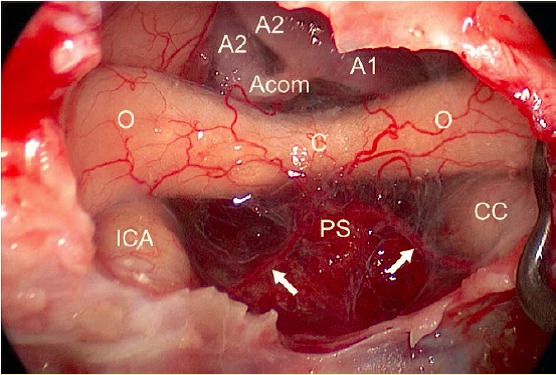
Endoscopic view with 45° optic on the suprasellar neuro-vascular structures (arrow: tractus hypophyseus superior)

Special attention must be paid to the vascular complex in the area of the first 2 segments of the anterior cerebral artery (**A1, A2**) with the anterior communicating artery (**ACoA**) (Figure 4 (Fig. 4)). In 60% of the cases, the artery “ACoA” reveals anomalies. Generally numerous, very small (>0.2 mm) but functionally very relevant branches depart in direction of e.g. the chiasm, the hypothalamus, and the subcallosal area [207]. Bigger cortical branches in the further course of the anterior cerebral artery are the fronto-orbital artery and the fronto-polar artery; another relevant branch is Heubner’s artery (arteria recurrens Heubneri) [151], [208], [209], [210].

The **cavernous sinus** extends over about 20x10 mm in anterior-posterior direction from the superior orbital fissure to the petrolingual ligament at the petrous apex [202], [211], [212]. The medial wall of the sinus consists of a very thin sellar and a sphenoid part. Planar processes of the venous sinus accompany the carotid artery also in the clinoid part [185], [204], [213]. The convergence and divergence of the brain nerves (III, IV, V_1_, VI) forms different triangles or S-shaped structures [155], [185], [213], [214]. The majority of the nerves is located in the lateral wall; a merely intracavernous course is only found of the abducens nerve (VI) and the plexus of the sympathicus around the internal carotid artery [185]. The lateral wall of the cavernous sinus with the here located brain nerves cannot be completely exposed by transnasal endoscopy without medial displacement of the internal carotid artery [196]. The lateral wall of the sinus consists of an external (meningeal) and an internal (periostal) layer; the medial wall has only one layer lateral of the very thin enveloping membrane of the pituitary gland [215]. The pituitary gland is surrounded by a connective tissue capsula that is firmly attached to the gland – at the outside there is a dura layer (partly as medial wall of the cavernous sinus) as well as sometimes periost [216]. The blood supply of the brain nerves originates from small branches of the carotid artery in its cavernous part [217], [218]. Anterior, there are relations of the cavernous sinus with the **orbitalis muscle** (Müller’s muscle) that may serve as landmark [148], [180], [181], [219]. The development of the venous sinuses with their intercavernous relations up to a “circular sinus” (in about 25%) is very individual. The venous intercavernous channels are always located apart from the free edge of the diaphragm. Their diameter measures about 3–5 mm. In dorsal direction, the venous plexus merges with the stronger venous plexus of the dorsum sellae and the clivus or dorso-lateral with the superior petrosal sinus [212], [220], [221].

At the frontal skull base, the subarachnoid space is crisscrossed by numerous arachnoid membranes and trabeculae. A particularly constant and conspicuous membrane is known as **“Liliequist membrane”** [222]. The subarachnoid space is enlarged between the membranes at many locations and forms a total of 9 **supratentorial cisterns**, anterior-inferior the **interpeduncular** and **pre-pontine** **cisterns** [223], [224]. A **“subdiaphragmatic cistern”** is located inferior of the sellar diaphragm and is in relation with the chiasmatic cistern [225]. Often tumors are in contact with the membranous borders of the cisterns; consequently, this knowledge helps looking for levels for dissection of tumor tissue. Meningeomas, however, grow regularly outside the cisterns.

The **neurovascular, suprasellar structures** can be subdivided referring to a virtual horizontal level through the inferior surface of the optic chiasm and a frontal level through the dorsum sellae. A supra-chiasmatic and a sub-chiasmatic compartment or a retrosellar and a ventricular segment result [226]. The optic chiasm itself can be located individually in anterior-posterior direction directly above the sellar diaphragm (usually) or above the tuberculum (“pre-fixed”) or the dorsum sellae (“post-fixed”) [154]. The relevant blood supply of the chiasm occurs via many small arterial branches mainly from cranial direction [227] (Figure 3 (Fig. 3), Figure 4 (Fig. 4)). 

**Figure 5:**
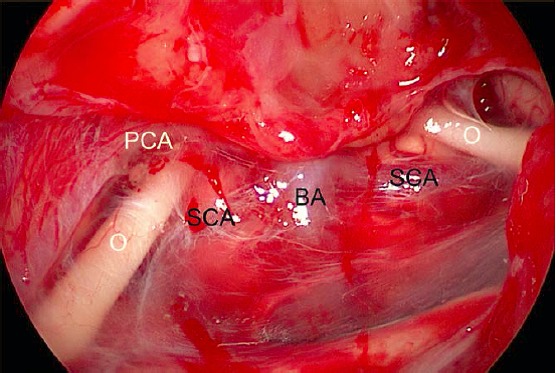
Endoscopic view with 45° optic into the interpeduncular cistern

The **oculo-motor nerve** (CN III) runs anterior of the midbrain first in the interpeduncular cistern (Figure 5 (Fig. 5)). Near the roof of the cavernous sinus it enters a short subarachnoid space, which is the oculomotor cistern of about 7 mm length. It can only be difficultly exposed by transnasal endoscopy. Afterwards, there is the petroclival and cavernous segment of the nerve in the lateral wall of the sinus, lateral to the internal carotid artery. Anterior, it passes the superior orbital fissure (fissural segment) [218], [228], [229].

The **trochlear nerve** (CN IV) passes posterior from the midbrain through 2 cisterns to the anterior tentorium (“cisternal segment”). By entering the tentorium, the “tentorium segment” and then the “cavernous segment” follow. In this area, the nerve is first right below the oculo-motor nerve (N. III) and lateral to the internal carotid artery. In the further course, it crosses N. III at the level of the “optic strut” and anterior it becomes the most superior neural structure of the cavernous sinus. When entering into the superior orbital fissure, a “fissural segment” can be discerned from the following “orbital segment” [230].

The **trigeminal ganglion** (CN V_1–3_) is located in the Meckel’s cave, the opening of which is directed to the posterior cranial fossa and is located inferior to the superior petrosal sinus, with a distance of 6.5 (4–8) mm from the entrance of the abducens nerve into the dura. There is a close spatial relation to the internal carotid artery and the cavernous sinus. From anterior, an accordingly narrow surgical access (“front door to Meckel’s cave”, see below) can be defined especially to the anterior-medial parts of the ganglion [101], [231], [232], [233]. The maxillary nerve is found about 7 mm supero-lateral of the sphenopalatine foramen. The length of the bony canal (V_2_) from the proximal to the distal opening (foramen rotundum) is estimated with about 12 mm [234].

The **abducens nerve** (CN VI) can be divided into 5 segments at the length of its course: after the exit at the pontomedullary sulcus, there is the cisternal segment up to the entrance into the dura about 17 mm below the posterior clinoid process, in a distance of 6–12 mm from the entrance point of the trigeminal nerve. The following part is a neural segment between the dura and the periost of the clivus in the middle of the venous spheno-petroclival plexus inferior of the petrosphenoid ligament (**“Gruber’s ligament”**) with a course through **“Dorello’s canal”** measuring about 9.5 mm. At the end of the canal, the nerve enters into a close anatomical contact with the sympathetic plexus via the lateral wall of the cavernous internal carotid artery and with the medial wall of Meckel’s cave. Dorello’s canal marks the transition of the superior to the middle third of the clivus, in the view from the front it is usually located about 10 mm below the level of the sellar floor. In the following “cavernous segment” the abducens nerve sometimes has a close relation to the lateral wall of the internal carotid artery. It runs medial to the ophthalmic nerve (N V_1_) to the transition of the “fissural segment” in the area of the superior orbital fissure and the annulus of Zinn. Anterior, there is the last part, the “intraconal segment”. The nerve is fixed with connective tissue in the area of the entrance into the dura and in the area of the petrous apex. This circumstance together with the relation to the carotid artery causes a high sensitivity of the nerve regarding direct compression and stretching [235], [236], [237], [238], [239], [240], [241], [242].

The **“Vidian nerve”** (nerve of the pterygoid canal; segment of the superficial petrosal nerve) can be identified easiest in the transition of the lateral floor of the sphenoid sinus to the medial lamella of the palatine bone [243]. In the frontal level, the letter “H” is marked by the medial pterygoid processes and the paraclival internal carotid artery of both sides and the floor of the sphenoid sinus lying in-between. The ostium of the canal of the Vidian nerve (pterygoid canal) has a diameter of nearly 3 mm, it is found in the transition of the horizontal into the vertical part of the “H”. The opening is located about 2–5 mm lateral of the anterior opening of the palatovaginal canal (palatosphenoid canal), about 5 mm lateral of the suture between vomer and sphenoid, and 5–9 mm infero-medial to the foramen rotundum. The last-mentioned distance correlates with the pneumatization of the sphenoid sinus. The canal measures around 14 (10–23) mm in length, the nerve itself has only a diameter of about 1 mm [84], [243], [244], [245], [246], [247], [248], [249]. The blood supply of the nerves occurs mostly from the maxillary artery with an artery of the pterygoid canal. In 30% of the cases, the accompanying artery is supposed to originate directly from the internal carotid artery [247], [249]. However, the general rule to identify the Vidian nerve by using the letter “H” is not always reliable [206]. In 65% of the cases, the nerve is located inferior of the floor of the sphenoid sinus and only in 28% it is found in the area of the floor [247]. Other authors expect a natural exposition of the nerve at the floor of the sphenoid sinus in the majority of the cases and in more than 2/3 of the cases a position medial of the medial plate of the pterygoid process [249].

**Figure 6:**
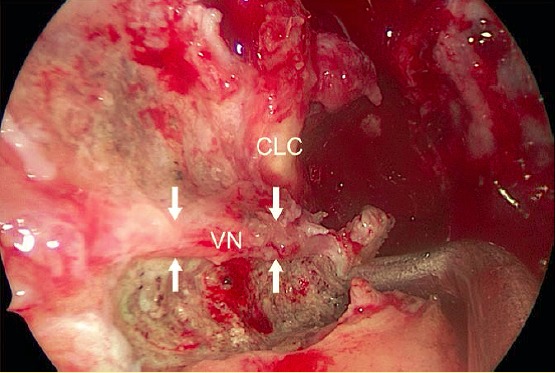
Endoscopic view with 30° optic on the course of the Vidian nerve of the right side (arrows)

Alongside the Vidian nerve (a.-p.) with its small accompanying artery, the second “knee” of the internal carotid artery may be encountered, i.e. the transition of the carotid artery from its horizontal course in the petrous bone to the vertical paraclival segment [39], [200], [243], [250] (Figure 6 (Fig. 6)). The paraclival carotid artery is located medial of the canal of the Vidian nerve, the horizontal part of the artery is superior and lateral to this canal [144]. Postero-medial of the nerve, there is the foramen lacerum, postero-lateral there is the mandibular nerve, infero-medial there is the Eustachian tube, and anterior the pterygopalatine fossa [251]. As rule (“clock concept”) it is generally accepted to first remove bony structures inferior-medial and then inferior-lateral of the Vidian nerve, if needed (on the left side: procedure in counterclockwise direction from 9:00 to 6:00, and then to 3:00) – the nerve directs the surgeon in dorsal direction to the horizontal part of the internal carotid artery in the petrous bone [250], [252], [253], [254], [255]. The recommendations slightly vary in their detailed formulation [249], [250].

The **“pterygopalatine fossa”** measures about 9 mm in depth (5–13 mm) (the cranial part is deeper than the caudal one). It consists of an anterior and a posterior compartment. The anterior one has distal parts of the maxillary artery with its branches, embedded in fat tissue (especially: posterior-superior alveolar artery, infraorbital artery, artery of the pterygoid canal, descending palatine artery, pharyngeal artery, terminal: sphenopalatine artery) [243], [248], [256]. The posterior compartment contains the neural elements (pterygopalatine ganglion with its Y-shaped afferent and efferent nerves; maxillary nerve). The maxillary nerve slightly descends distal of the foramen rotundum [257]. In the course of the infraorbital nerve, it marks the sagittal level where the pterygopalatine fossa merges in lateral direction via the pterygomaxillary fissure into the infratemporal fossa [251], [254], [258], [259], [260], [261]. The Vidian nerve enters into the fossa via the pterygoid canal, infero-medial to the maxillary nerve [254]. Between the Vidian nerve and the maxillary nerve, a corridor to the lateral wall of the sphenoid sinus can be created by removing bony structures [258]. A dissection of terminal branches of the maxillary artery presents: the infraorbital artery, the descending palatine artery, the Vidian artery, the sphenopalatine artery, the posterior nasal artery, the pharyngeal artery in the palatosphenoid canal [243], [262].

Medial, the **“infratemporal fossa”** is in contact with the pterygopalatine fossa and postero-medial with the parapharyngeal space. It is located below the surface of the greater wing of the sphenoid bone, dorso-lateral of the maxillary sinus, lateral of the lateral pterygoid process, medial of the mandibula, and in front of the styloid process with its muscle insertions, carotid artery, and deep parotid lobe. It contains among other structures the proximal 2 segments of the maxillary artery (retromandibular and intramuscular segments), the mandibular nerve (N. V_3_), the pterygoid muscles, the temporalis muscle, the otic ganglion, the deep part of the parotid gland, and an extended venous plexus. An important landmark is the lateral process of the pterygoid: If a prolongation of the posterior edge of the lateral process is imagined in dorsal direction, the foramen ovale is encountered; in a small distance, the foramen spinosum follows postero-laterally. The parapharyngeal internal carotid artery is located about 2 cm dorsal in about the same sagittal level. The distance of the foramen ovale to the anterior nasal spine amounts to around 8 cm. Beside the lateral process, the buccal nerve may serve as guideline for identifying the foramen ovale [36], [146], [257], [263], [264], [265], [266], [267], [268]. Dorsal of the foramen ovale, the internal carotid artery is found in a distance of about 5 mm in the middle part of its petrosal segment [269]. The dimension of the foramen ovale amounts to about 8×5 mm [270], the distance of the posterior edge of the lateral process is about 6 mm. The distance of the posterior wall of the maxillary sinus measures around 18–20 mm [268]. Behind the lateral pterygoid muscle, the tensor muscle and the levator veli palatini muscle are located medial in close neighborhood of the Eustachian tube [271]. The mandibular nerve is located directly in front of the tube [162]. The distance from the isthmus of the Eustachian tube to the proximal aperture of the carotid canal amounts to only about 5 mm [272]. The foramen spinosum and the foramen ovale are both only 4 (2–6) mm away [232]. 

Posterior and medial, the **“parapharyngeal space”** merges to the retropharyngeal space. Medial, it confines to the superior pharyngeal constrictor muscle, lateral to the pterygoid muscles and the parotid gland (infratemporal fossa). Superior it leads to the skull base; in caudal direction, it is bordered by the styloid muscles, the submandibular gland, and the mandibulo-styloid ligament. In reference to a styloid “diaphragm” consisting of muscular parts (posterior digastric muscle, stylohyoid muscle, stylopharyngeal muscle) and a stylopharyngeal aponeurosis, a pre- and poststyloid compartment can be differentiated. The latter contains the carotid artery, the internal jugular vein as well as the inferior cranial nerves IX–XII [146], [152], [243], [252], [264], [273]. The most important landmark for the preparation of the poststyloid compartment is the Eustachian tube. Important landmarks for the parapharyngeal carotid artery are: the foramen ovale directly anterior of the poststyloid compartment, the styloid process, and the transition of the cartilaginous to the bony part of the Eustachian tube with the superior points of insertion of the levator veli palatini muscle [146], [252].

Anatomical classification systems of the literature, which have been mentioned selectively are summarized in Table 7 (Tab. 7). 

### 2.3 Structural quality and process quality

#### 2.3.1 Preparation of the patient

The general recommendations of perioperative care during rhino-neurosurgical interventions are based on well-known standards of neurosurgery and endonasal sinus surgery [274]. Preoperatively, the patient is intensively informed about the indication, therapeutic alternatives, performance, complications, and sequelae of the planned intervention. Among other aspects, also unforeseen but necessary expansions of the endonasal intervention and also putative, forced intraoperative changes of general concepts going over to conventional approaches (e.g. open craniotomy) are mentioned [2], [275], [276], [277].

Preoperatively, a thorough ENT specific examination is performed with endoscopy of the inner nose. Attention is paid to spatial dimensions in the respective surgical corridor or individual narrow passes or mucosal infections. Further aspects are local particularities as for example residual conditions after previous surgeries or ridged septal spurs that may hinder the creation of naso-septal flaps [278].

Depending on the individual clinical appearance of the patient, the respective findings, and the planned intervention, further measures should be performed:

- Neuro-ophthalmologic examination with determination of the visual acuity and visual field testing [92], [279], [280], [281], [282], [283].
- Endocrinological examinations (serum cortisol, plasma ACTH, ADH, TSH, thyroxin or estradiol, prolactin; serum-sodium; urine – specific weight) [18], [92], [279], [280], [283], [284].

Patients suffering from active infection of the paranasal sinuses should undergo antibiotic therapy for 10–15 days preoperatively [285].

#### 2.3.2 Imaging

Generally, rhino-neurosurgical interventions are enabled and safeguarded by advanced imaging. Instructions and preconditions for input of the imaging data into navigation systems have to be respected. Technical details are handled in different ways – studies on the evidence are not possible in this context [286].

Routinely performed interventions at the pituitary gland are secured with a special MRI of the sella [280]. More advanced and extended interventions require a high-resolution multiplanar CT scan as well as MRI, possibly with later CT-MRI fusion in combination with angiography [2], [36], [55], [61], [106], [286], [287], [288], [289], [290]. In the MRI, the following aspects must be observed: landmarks, liquor space (T2) between lesion and brain, size of the lesion, location with regard to big vessels and optic nerves, potential displacement of structures, or fibroses [111].

Fusioned angiography facilitates the navigation of bigger vessels [291], [292] and helps excluding anomalies as for example a primitive trigeminal artery [293]. Optimized MRI protocols, e.g. with CISS sequences should be especially helpful for visualization of the anatomic border region of the anterior skull base [294]. The individual consistency of a tumor, however, can only be predicted with great difficulties by means of imaging techniques [112].

In case of malignomas, sonography of the cervical soft tissues is performed preoperatively to exclude regional metastases. According to the literature, even preoperative PET-CT of the whole body is recommended [32], [69], [126], [143].

In the individual case, also intraoperative imaging by means of CT scan or MRI could be helpful. Displacement of tissue parts or developing complications in the depth could thus be identified [2], [290], [295]. Intraoperative CT scan is especially useful in interventions of bony structures of the cranio-cervical transition, intraoperative MRI may be appropriate e.g. for clivus chordomas [66], [296], [297].

Interventional neuro-radiologists are part of the therapeutic team and should be available peri- and postoperatively at short term [298]. If the tumor is well vascularized, preoperative embolization is recommended [32], [55], [76], [93], [267], [299], [300]. If intraoperative injury of the carotid artery can be anticipated, carotid occlusion testing is performed [90]. In single cases, self-expanding intravascular stents have been inserted into the parapharyngeal petrosal course of the internal carotid artery as prophylaxis in order to protect against iatrogenic lesions (“Solitaire AB neurovascular remodeling device, ‘ev3 Inc.’”) [143].

#### 2.3.3 Pre-medication

In the literature, pre-treatment with oral steroid and antibiotic (third generation cephalosporin or amoxicillin clavulanate) is recommended [9], [301].

As pre-medication, diphenhydramine (50 mg i.v.) and cortisone (e.g. dexamethasone 10 mg i.v.) are applied in combination with fluorescein injection [41], [82], [92], [93], [286], [302], [303]. Fluorescein application is performed via lumbar puncture, mostly as 0.2 ml 10% fluorescein in 10 ml CSF [82], [302]. For local care of the nasal mucosa, in single cases, streptomycin or neomycin nasal spray has been applied for two days before the intervention [301], [304].

#### 2.3.4 Positioning

The general direct preparations of an intervention correspond to the ones of routine endonasal sinus surgery [101]. The majority of surgeons prefer fixation of the head in a Mayfield clamp [41], [101], [104], [168], [302], [303], [305]. Other surgeons intentionally do without such fixation [18], [93], [163], [306], [307].

The upper part of the patient’s body is lifted of 5–15° (sometimes even 30°) [61], [98], [163], [308], in cases of interventions on the pituitary gland alternatively a slight Trendelenburg position is described [18]. According to the location of the target region, the head is held or fixed; interventions at the anterior skull base require significant extension (10–30°), posterior-inferior lesions often need flexion (20–40°). Additionally, the head is turned slightly (10–15°) in direction of the surgeon and for right-handed surgeons it is put to the left of 10–15° in the longitudinal axis of the body [37], [41], [94], [98], [163], [168], [302], [305], [306], [308], [309], [310].

The periumbilical region and the hip of the patient are prepared for fat or fascia harvesting [82], [93], [101], [302], [303]. A bladder catheter is inserted and if needed a central venous and an arterial line is provided [41], [82], [93], [302]. The intra-arterial pressure is recorded. Often controlled hypotension is not recommended, normal tension or a slightly hypotensive condition are intraoperatively appropriate (MAP >85 mmHg) [69], [301].

Depending on the intervention and the risk for critical neuronal structures, it is recommended to provide neuromonitoring (somatosensory evoked potentials, brain stem potentials, spontaneous or triggered EMG of muscles of the cranial nerves) [32], [36], [55], [69], [104], [113], [290]. Interventions at the inferior clivus are controlled for example via EMG of the cranial nerves VI, IX, X, XI, and XII [164], [295]. Persisting intraoperative changes of somato-sensory evoked potentials are predictive for postoperative neurological deficits [311].

In single cases, additionally the regional cerebral oxygen saturation was measured (Somanetics INVOS Oximeter) [309].

Some authors recommend the preoperative intrathecal fluorescein application in order to control later reconstruction of the skull base [92], [93], [118], [286], [312], [313].

#### 2.3.5 Local measures

The outward skin of the face, nose, and upper lip is washed with polyvinylpyrrolidone (PVP) iodine solution [37], [55], [168].

The nose is treated or rinsed with PVP iodine solution soaked gauze [37], [102], [205], [314], [315]. Alternatively, the nose is treated or rinsed with 5% chlorhexidine or antibiotic solution (e.g. clindamycin) [18], [280], [308], [316], [317], [318]. Other authors do not use antiseptics in the inner nose because no preventive effect was observed with regard to postoperative infections [41], [310]. Decongestion of the nasal mucosa was achieved by topical application of epinephrine (mostly 1:1000) or oxymetazoline, sometimes combined with infiltration of the mucosa [41], [61], [205], [283], [301], [319], [320].

#### 2.3.6 Instruments, preparations for anesthesia, monitoring

Rhino-neurosurgical interventions can only be performed with adequate instruments. Endoscopes with different angular optics (0°, 30°, 45°, 70°) are essential as well as HD video chains connected to several monitors [26], [31], [93], [98], [302], [321], [322], [323]. Endoscopes with a diameter of 4 mm are usually applied, in single cases for children also 2.7 mm in diameter [132], [137], [308], [324], [325], [326]. Mostly used is the 0° optic followed by 30° endoscopes [168], [327]. Angular optics allow the view into the lateral recess of the sphenoid sinus [328], [329], the field of view in transclival approaches can be enlarged [330] and probably the hypothalamus, the 3^rd^ ventricle, and also the lateral ventricle can be examined [61]. Stereo endoscopes or endoscopes with 5 mm in diameter are currently not routinely used [316], [331].

Many surgeons use special, e.g. pneumatic flexible supporting arms for the endoscope as “third hand” [18], [82], [98], [305], [306], [330], [332], [333], [334], [335], [336]. Others only use such devices in single phases of surgery or for selected interventions such as surgery of the pituitary gland [92], [93], [279], [308], [324]. However, many surgeons do explicitly without supporting device emphasizing a more effective way of working in difficult situations (e.g. bleeding) [55], [61], [168], [283], [337], [338]. Furthermore the supporting arm for the endoscope eliminates one very important characteristic of the endoscope, which is the dynamic field of view [27]. 

Endoscopes may be coupled with a rinsing or rinsing and suction system [18], [41], [55], [104], [279], [168], [280], [308], [318]. According to other authors, an endoscope with rinsing system is generally less manageable. They prefer sprinkling the endoscope manually from exterior [61], [90], [283].

The following necessary instruments are mentioned in literatur:

- Several microinstruments with longer shafts, if needed with bayonet handles [146], [163], [290], [336], [339].
- According instruments for bipolar coagulation, if needed with pistol grip and different, possibly exchangeable tips [36], [55], [102], [111], [302], [336]. Monopolar electro-surgical instruments are used as needle, ring, or suction coagulator [82]. However, they are no longer appropriate apart from the proximal surgical corridors [290].
- High-speed drills with extra-long shafts and with straight and angled drill attachments at the tip [55], [66], [92], [115], [163], [262], [290], [318], [337].
- Ultrasound dissector for preparation of soft tissue with simultaneous protection of neighboring vessels [55], [82], [104], [302], [337], [340]. However, attention must be paid to heat development at the tip of the instrument regarding delicate neighboring structures [290].
- It may be useful useful to apply a special ultrasound curette “Sonopet” [341].
- Microdebrider/shaver for debulking and removal of solid tumors [104], [107], [336], [342], [343].
- Micro-Doppler probe for identification of arterial vessels [118], [142], [286], [309], [313], [344].
- Laser systems. Diode laser may be applied to mark the resection margins [9], [126] and CO_2_ laser in transsphenoid interventions for thinning out bony structures, dura opening, posterior septectomy and sphenoidotomy, incision of pituitary adenomas [345].
- The use of a cell-saver was discussed in cases of expected high blood loss – however, the security of the system with regard to contaminations is not proven [346].
- If instruments or parts of technical systems break down, a replacement equipment has to be ready [347].
- Flexible or controllable instruments [330].
- Suction devices, if needed with flexible tips. It is reasonable to use 2 suction devices simultaneously [37], [55], [64], [72], [348].
- Medical consumables for reconstruction of the dura and hemostasis, vascular clips [36], [267].

Even if single transsphenoid routine interventions are sometimes performed without navigation system [319], [322], the use of those systems is generally urgently indicated. The intraoperative displacement of tissue structures (brain shift) is low in structures and lesions of the skull base and thus it is not an adverse factor [30], [36], [55], [104], [111], [283], [349]. However, a deviation of 0.8 to 2 mm must be expected [296], [350].

**Figure 7:**
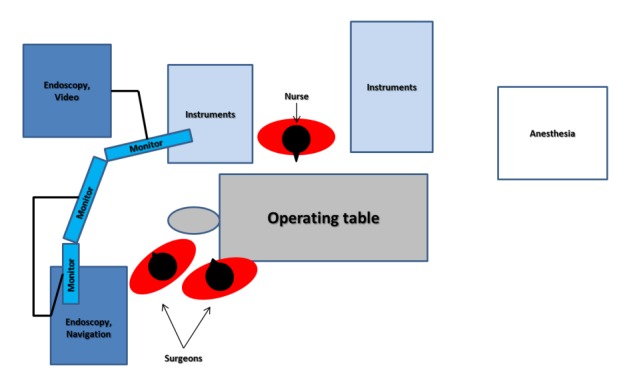
Arrangement of devices and personnel in an operation theater for a rhino-neurosurgical intervention (4-hands technique) [111]

Various arrangements of actors, monitors, and equipment are possible [310]. Generally, each surgeons has to have a monitor in front of his eyes. A third and if needed a fourth monitor is used by the nurses, the anesthesiologist (also for neuro-monitoring) and possible observers [41], [290]. The surgeons stand on the right side of the patient [55]. The height of the table and the arrangement of the instruments have to be based on ergonomic principles [351]. The positioning of the devices and the personnel during rhino-neurosurgical interventions is illustrated in Figure 7 (Fig. 7). 

#### 2.3.7 Process quality

Routine transsellar interventions at the pituitary gland are generally performed by neurosurgeons without the support of ENT specialists [98]. For rhino-neurosurgical interventions, however, an interdisciplinary approach is urgently recommended: The team consists of an ENT specialist who is experienced in endoscopic sinus surgery and a neurosurgeon with special expertise in the field of surgery of the pituitary gland and transcranial interventions at the skull base. The need of advanced expertise of both partners is justified by the fact that the interventions may have to be changed intraoperatively from endonasal to open approaches if required. Both surgeons should be involved in all decisions and measures [30], [183], [352]. They may be regarded as the core unit and heads of an enlarged interdisciplinary skull base team, which means that they work in close cooperation with an ophthalmologist, radiotherapist, hemato-oncologist, interventional radiologist/neuro-radiologist, and neuropathologist [2].

Rhino-neurosurgical interventions represent a particular challenge: in general the durations of surgery are much longer than of traditional surgeries via craniotomy, a duration of 6–10 hours may be expected [100]. In extreme cases, the intervention has to be performed in several sessions because of time reasons (i.e. because of a protracted and high blood loss or restriction of reserves in terms of staff) [36], [69], [353]. Regarding the mentioned surgeries, there is a longer learning curve [320], [354]. Effects of this curve are not only positive regarding a shorter duration of the intervention [120] but also with regard to a clear improvement of the surgical results [26], [129], [355]. The good results of rhino-neurosurgical interventions in case of malignomas are only achieved by experienced surgery teams [96]. The rate of for example complete tumor resection (GTR) and a reduction of postoperative diabetes insipidus, postoperative problems of vision, or postoperative CSF fistulas may be encountered with the gain of experience. The surgical results reach a positive level after about 20–50 cases [124], [318], [338], [356], [357], [358]. Regarding transsphenoid surgery of the pituitary gland, however, it is generally expected that the complication rate is only reduced significantly after 200–500 interventions [359]. 

The mentioned facts promote the argument to develop an intensive and constant training program for interested surgeons or surgical teams [54], [129] and to establish a special program for quality management in according centers [360]. The objective of those trainings is not only the individual development of skills but also cooperation abilities: a professional teamwork is particularly important in cases of undesired events such as for example bleedings [338].

At the beginning, there should be a training with anatomical specimens and models. Microanatomical exercises with non-fixed specimens are strongly recommended to all future rhino-neurosurgeons. Required equipment and devices for such labs are given [361]. Special training programs for preparation courses have been published [e.g. [44], [55], [70], [362]. For accompanying virtual training, computer-assisted 3D models for visualization of surgical planning are at disposition [363], [364], [365]. 

Regarding the first practical training steps, a simple abstract plastic model of endoscopic surgery of the pituitary gland has been developed [366]. An in-vivo animal model of pigs for endoscopic surgery of the pituitary gland has been described [367] as well as an in-vitro model of a sheep head for training of endoscopically assisted performance of naso-septal flaps [368] and an abstract in-vivo animal model of the rat for basal endoscopically controlled preparation techniques [369]. For training of more advanced skills, a complex model of anatomical human fresh specimens with simulation of tumor disease by injection of polymers has been established [370], [371]. An alternative model uses special animal specimens (“chicken wings”) in combination with an alloplastic sinus model for team training of microsurgical manipulations in “2-minds-3/4-hands” technique [372]. Basically similar is the combination of 3D silicone models with biological material (eggs) [373]. Another complex in-vivo animal model of a dog for surgeries in the area of the cavernous sinus works with implantable tumor cells [374].

The training with models is appropriate with the background of the experience that the necessary forces of manipulations of soft tissue with conventional instruments during interventions (e.g. of the pituitary gland) are very low – and that simultaneous manipulations of neighboring bones often require the twentyfold amount of power [375]. Effectivity of abstract exercises for clinical practice are not yet proven, however, the hope is justified that the individual learning curve can be reduced [370], [372].

For hospital practice, a gradual training plan of increased difficulty levels has been elaborated (Table 8 (Tab. 8)). It is suggested that each surgeon should have performed about 20–30 interventions of a certain complexity level before addressing the next difficulty level [376]. Other authors recommend 30–50 interventions of the pituitary gland as primary precondition before interventions of higher difficulty degrees are assessed [354]. In this context, first interventions are performed of hormonally inactive pituitary adenomas, older patients with wide noses and good pneumatization [347].

The surgeon’s experience and the size of the institution have a relevant impact on the result and the treatment costs [377]. With this background, subspecialization of the physicians was recommended on the one hand [357]. On the other hand, the complete range of rhino-neurosurgical interventions should only be performed in centers with a minimum occurrence of such cases [336], [378], [379]. The rate of postoperative CSF fistulas for example is lower in bigger centers than in smaller departments [380]. For compensation, a mentoring program with regular board discussions, phone conferences, and common examinations was offered by bigger centers [381].

## 3 Basic surgical techniques, hemostasis, special technical aspects

The historical beginning of advanced transnasal surgery was with one surgeon and the access via one nasal cavity using an endoscope-holder and C arm for intraoperative radiography [316], [331].

In the interdisciplinary rhino-neurosurgery, however, the management has completely changed: the whole interventions are performed by two experienced surgeons of both disciplines (ENT, neurosurgery) (“2 surgeons, 3 to 4 hands”; “4 hands – 2 nostrils”; “2 nostril bimanual technique”; “binasal/binostril/binarial approach”) [26], [61], [64], [83], [102], [104].

Right-handed surgeons insert the endoscope via the right side in cases of appropriate anatomy and target structure. A 0° optic is positioned in the nostril expanded in cranial direction at 12 o’clock position, the suction at 6 o’clock position – if the 30° optic is used, it is the other way round. Further instruments are positioned via the contralateral side. In the context of “3 hands technique”, one surgeon holds the endoscope, the other one works for example with suction and instrument. Using the “4 hands technique”, the first surgeon takes the endoscope and the suction device. Alternative handlings are known, as for example the left-sided or transseptal insertion of the endoscope [119], [382].

Manipulations in the posterior parts of the nose are facilitated by the “4 hands technique via both nostrils” by resection of the posterior nasal septum. It is recommended to resect 1–2 cm (sometimes even 3 cm) of the dorsal septum. Often at the beginning the total resection of the ipsilateral middle turbinate with lateroposition (rarely also resection) of the contralateral middle turbinate is done due to the same reasons. If needed, even ethmoidectomy with (partial) resection of the superior turbinate must be performed or a partial removal of the medial pterygoid process or partial resection of the posterior inferior turbinate. In contrast, suitable cases are limited to lateralization of the turbinates followed by covering with gauze, e.g. in children. In transsphenoid or transtuberculum-transplanum interventions, mostly lateralization of both middle turbinates is sufficient, if needed with unilateral resection of the middle turbinate on the side where the endoscope is positioned. From the septum, only the rostrum has to be removed. A similar approach is described for the exposition of the upper third of the clivus. If a naso-septal flap is needed, the posterior septectomy is performed only after elevation and temporary displacement of the flap (see below) – a prophylactic cauterization of the sphenopalatine artery must be avoided [37], [41], [44], [66], [70], [94], [101], [102], [116], [117], [119], [168], [183], [282], [283], [296], [307], [315], [321], [326], [334], [383], [384], [385], [386], [387], [388], [389], [390], [391], [392]. In single cases, it may be helpful to perform first steps such as for example posterior septectomy and sphenoidotomy only in 2 hands technique over one side of the node (“mononostril”) and then to proceed to 3 or 4 hands technique (“binostril”) [27], [319].

### 3.1 Division of labor

Based on the literature, the division of work in rhino-neurosurgery is asymmetric: the ENT surgeon is mainly responsible for the direct extracranial access with exposition (“nasal and paranasal phase”) and for parts of the reconstruction, e.g. by preparing a naso-septal flap. The rhinosurgeon may even perform the initial steps of the “nasal phase” alone. The bimanual ablative intradural surgery in the proper sense, however, is performed by the neurosurgeon [93], [168], [205], [298], [309], [390], [393]. The role of the ENT surgeon in the second intracranial phase is described in the literature as “navigator”, “assistant”, “endoscopist”, or “endoscope operator” [44], [319], [394]. Due to experienced navigation of the endoscope, the “navigator” allows having the plastic impression of the anatomical target and neighboring structures, the clear view (with or without simultaneous suction) with maintained degrees of freedom for using other instruments.

### 3.2 Preparation technique, surgical strategy

In general, intradural dissection follows a step-by-step process for tumor surgeries:

1. Peripheral devascularization of the tumor in the surgical corridor, followed by internal debulking.
2. Definition of the gap between the peripheral tumor capsula and the surrounding neuronal tissue; mobilization of the tumor capsula.
3. Extracapsular dissection and isolation of neurovascular structures.
4. Resection of the tumor capsula.

Blunt preparation with gripping and drawing of intradural tumor parts, e.g. by use of forceps, is avoided if possible. Fine preparation is rather performed with the suction device applying reduced suction power. The suction instrument is held with the non-dominating hand; it stabilizes the tumor tissue during the mainly sharp preparation or manipulations with the dissector. The view is improved for example by rinsing with physiological saline solution. If rinsing is performed with light pressure, the tissue is forced apart and inspection is easier (“hydroscopy”, “diving technique”) [81], [325], [395]. Another simple instrument supporting the exposition is the “q-tip retractor” [396]. In cases of relevant bleeding, the view must be maintained by inserting a second suction device [55].

Generally, a complete tumor resection is the objective. In particular, the tumor insertion zone is concerned in this context. Under certain circumstances, however, this objective has to be left because of several possible reasons: tumor parts cannot be accessed, invasion or firm adhesion of the tumor to delicate structures (among others hypothalamus, thalamus, fornix, basilar artery, carotid artery, anterior communicating artery, optic nerve, chiasm) [90], [355]. En bloc resection is not necessary [37], [64], [69], [105], [113], [168], [183], [315], [338], [397], [398].

### 3.3 Hemostasis

Measures for hemostasis during nasal and paranasal (extradural) phases of preparation correspond to usual practices of sinus surgery [3], [11], [399]. Generally, a distinction may be drawn according to the bleeding source (artery, vein) and the bleeding quality (low-flow, high-flow).

In the context of rhino-neurosurgery, the following possibilities of hemostasis must be considered:

- Bipolar coagulation. For interventions in hidden areas of the internal nose, coagulation forceps with different tips are used, if needed with pistol grip. Monopolar instruments should not be applied within the sphenoid sinus, at the skull base, or intracranially. In general, certain bleeding sources such as for example the clival venous plexus, cannot be treated sufficiently by coagulation [163].
- Irrigation with warm physiological saline solution. An optimal temperature of 40°C is mentioned, but also lower temperatures are effective.
- Hemostatic materials for topical therapy especially of venous bleeding:
  - Gelatin-based hemostatics in different compositions (e.g. “Surgifoam^®^”, “Gelfoam^®^”).
  - Combinations of gelatin-based products with thrombin preparations (e.g. “FloSeal^®^”, “Gelfoam plus^®^”, “Surgiflo^®^”).
  - Micro-fibrillary collagen (e.g. “Avitene^®^”)
  - Sponge made of plant-based poly-N-acetyl-glucosamine (e.g. “Syvek^®^”).
  - Collagen sponges (e.g. “Lyostypt^®^”), partly with coating of fibrinogen or thrombin fleece (e.g. “Tachosil^®^”, “TachoComb^®^”).
  - Net or gauze made of oxidized cellulose (e.g. “Equitamp^®^”, “Surgicel^®^”, “Tabotamp^®^”).
  - Fibrin glue with autologous (e.g. fascia, muscle) or xenogeneic tissue.

A so-called “sandwich” (peripheral layer of neuro-gauze, central layer of hemostatic material) is effective. It is applied specifically and held on the bleeding for several minutes with appropriate pressure. For local insertion of the material, special instruments are suggested [400].

Intradurally placed tamponades may be dangerous because they allow continued or delayed bleeding below the material in direction of the endocranium.

Generally, case reports must be considered about rarely occurring severe complications (pulmonary thromboembolism by washing away of material) caused by the application of gelatin-based preparations with thrombin during other neurosurgical (spinal) interventions [401], [402].

- Bleeding from the bone is stopped with the diamond drill without local rinsing or by applying bone wax.
- For prevention, a highly vascularized tumor should be embolized. Alternative measures of preventive devascularization are the prophylactic coagulation for example of ethmoid vessels or the sphenopalatine artery and non-specific afferent vessels of the periphery of the tumor. Functionally unimportant vessels or e.g. intercavernous sinuses may also be closed with vascular clamps.

The treatment of severe arterial bleeding requires a coordinated cooperation of both surgeons. The proximal carotid artery can be prophylactically exposed and “secured” in the paraclival area or in the neck in interventions where respective complications must be expected. After coagulation of big arterial vessels, the development of secondary aneurysms must be excluded by subsequent examinations [2], [26], [36], [41], [55], [69], [92], [168], [290], [309], [313], [322], [330], [403], [404], [405], [406], [407].

### 3.4 Transethmoidal/transcribriform interventions

Endoscopic endonasal tumor surgeries with their anatomical basics are generally known to ENT surgeons as part of advanced endonasal sinus surgery or in combination of endoscopic endonasal interventions with transcranial accesses [54], [408], [409], [410], [411].

In the specific focus of rhino-neurosurgical interventions, there are extended malignomas with massive infiltration of the dura (“transcribriform craniectomy”), meningiomas, olfactorius schwannomas or large meningo-encephaloceles, dermoid cysts or fistulas [7], [9], [302], [314], [320], [348], [412], [413]. According to the literature, endonasally treated malignomas are mostly relatively small – the surgical results are similar for those patients as for cranio-facial surgeries [54], [414].

In general, the endonasal masses of encephaloceles are removed and the stalk is coagulated and transsected [415].

Regarding malignomas of the paranasal sinuses with involvement of the skull base, a complete ethmoidectomy (corridor of the transethmoid partial access) with abrasion of the ethmoid foveae, is mostly combined with a resection of the turbinates, followed by exposition and the removal of the lamina cribrosa (corridor of the transcribriform partial access) as well as resection of the cranial nasal septum. If needed, the intervention is planned bilaterally and maximized. Anterior, a prophylactic type III drainage of the frontal sinus is performed. In the context of those measures, first the exophytic intranasal parts of the tumor are resected. The anterior and posterior ethmoid vessels are identified, prophylactically coagulated, and transsected. Measures at the exposed dura and intradurally are performed only then – in this way, a bleeding and intracranial tumor dissemination is avoided. In typical cases, the dura is pushed away from the crista galli and the anterior bony attachment of the crista is abraded. The local falx cerebri has to be identified, electrosurgically treated, and transsected. After total exposition of the dura “in sano”, the meninges may be incised around the focus and the specimen can be moved gradually in caudal direction and resected. The olfactory nerves may have to be transsected inevitably [72], [107], [317], [411], [415], [416], [417].

Thus, all skull base structures from the caudal posterior wall of the frontal sinus with the crista galli to the sphenoid sinus, in the width from one lamina papyracea to the other can be exposed and resected if required. With an individual adaptation of the exposition, for example also encephaloceles, meningoceles, meningiomas, or esthesio-neuroblastoma are treated. One often inevitable consequence of extensive interventions is the removal of olfactory mucosa with a subsequently impaired or lost olfaction. The corridor through the anterior skull base has a depth of 29–40 mm in anterior-posterior direction, the width amount to around 20–27 mm over both sides [418]. An extension in dorsal direction is possible at the expense of the sphenoid planum [348]. Attention must also be paid to the cranial extension of the manipulations, the crista galli is about 13 mm high and 13 mm “long” [416].

If the described large defects are covered with soft tissue in several layers, there is no need to expect secondary sinking of the frontal brain or development of an iatrogenic encephalocele [419].

### 3.5 Orbital interventions

(Trans-)orbital interventions represent overlapping fields of “rhino-neurosurgical surgery” in their proper sense and interventions of the regular “advanced endonasal endoscopic sinus surgery”. Merging also occurs to transpterygoid interventions with target structures in the “sino-orbito-cranial interface” that may require a combination of transoral, transseptal, or prelacrimal approaches [181].

Selected foci (e.g. hemangiomas, schwannomas, meningiomas, bone-developing tumors, biopsies) in the intraconal tip of the infero-medial parts of the orbita are suitable for those approaches. The surgical corridor is mostly located between the medial and inferior rectus muscle. At the beginning, ethmoidectomy is performed with fenestration of the sphenoid sinus, followed by the removal of the dorsal lamina papyracea with incision of the periorbita. If needed, the medial walls of the superior orbital fissure or the canal of the optic nerve are removed; a greater range of action for 2 surgeons may be achieved by posterior septectomy (over around 2 cm). The medial rectus muscle and the inferior obliquus muscle can be fixed by threads or vessel loops from exterior or interior and drawn or even displaced in medial direction – very useful in the last mentioned case is a thread drawn through the nasal septum. In order to allow improved preparation by 2 surgeons, posterior nasal septectomy may be necessary. The preparation of the orbita is performed by means of gauze and blunt dissection. Finally, fat tissue is put over the exposed muscles to avoid extensive scarring. According to some reports in the literature, however, the complication rate (e.g. persisting diplopia) is rather high (10–20%) [26], [69], [127], [420], [421], [422], [423], [424], [425].

### 3.6 Transsellar interventions, advanced transsphenoid interventions

The transsphenoid corridor with a transsellar access is one of the most frequent rhino-neurosurgical interventions [426] that focus on intrasellar pathological processes. Smaller extensions of those lesions in suprasellar direction or in direction of the cavernous sinus can be controlled and treated with angular optics. In the majority of the cases, those are micro- and macroadenomas, intrasellar craniopharyngiomas, Rathke or dermoid cysts. If suprasellar structures are relevantly involved, a combination with a transplanum-transtuberculum intervention is required [427], [26], [60], [109], [280], [378], [428], [429], [430]. Another diction differentiates paraseptal, transethmoid-sphenoid, and transethmoid-pterygoid-sphenoid interventions [321].

The interventions generally consists of a “nasal phase” and a “sphenoid phase”, followed by a “sellar phase” and a “reconstructive phase” [431].

At the beginning, the anterior wall of the sphenoid sinus is exposed step-by-step. In cases of intrasellar lesions of limited dimension (adenomas of <2.5 cm), the accompanying interventions at the other paranasal sinuses etc. are minimized as far as possible [41]. In other cases, first a uni- or bilateral posterior ethmoidectomy is performed, with resection of (1–)2 cm of the posterior septum, if required [432]. Parts of the superior turbinate are often removed in the direct surgical corridor (i.e. on one side, mostly on the right) as well as a more or less large part of the middle turbinate. The remaining turbinates are lateralized. Bilateral maximal sphenoidectomy follows together with abrasion of the intrasphenoid septa leading to the complete exposition of the sella, the tuberculum septi, and the sphenoid planum. The use of the shaver in this phase is avoided [310]. In the sellar phase, the sella is opened and the lesion is resected gradually. The intervention is completed with reconstruction of the sella and reposition or medialization of the turbinates [168], [226], [308], [433].

The term of “extended/expanded” transsphenoid surgery is not consistently used in the literature. Generally it applies to all endoscopic “transtuberculum-transplanum interventions” of the skull base that aim at lesions outside the sella or extend from the sella to other structures. Less accepted is the mentioned term to describe a resection of the middle or superior turbinate with ethmoidectomy or posterior septectomy and an extended fenestration of the sphenoid sinus. Other authors use the term to focus on any “binostril approach” or specifically the opening of the tuberculum sellae or the sphenoid planum with exposition of parasellar structures or fenestration of the clivus (see below) [12], [93], [113], [282], [310], [387], [398], [434], [435].

In case of advanced interventions, the optic nerve and the paraclinoid carotid artery may be presented via the medial opticocarotid recess [72]. Via the dorsum sellae (“transdorsum approach”) the interpeduncular fossa and the respective cistern can be exposed if the pituitary gland is temporarily displaced. For this purpose, it is removed from its surrounding connective tissue and the inferior pituitary artery is transsected. Afterwards, the pituitary gland can be moved temporarily in suprasellar direction into the prechiasmatic cistern. After removal of the posterior wall of the sella and the posterior clinoid processes, the interpeduncular fossa is reached [205], [391]. However, this complete “pituitary transposition” bears the high risk of pituitary insufficiency so that it is better to only remove unilaterally the pituitary gland from its dural bed followed by mere medial displacement.

If the petrous apex is well pneumatized, a contact area to the sphenoid sinus is found of about 11 mm in diameter [166]. Here for example an expanding cholesterol granuloma may protrude into the sphenoid sinus. The transsphenoid fenestration of such cysts is comparably uncomplicated. In other cases, a combined transsphenoid/transpterygoid intervention has to be performed in order to achieve permanent drainage of the cyst (see 3.9). For support, in individual cases a drainage tube is inserted for 2–5 months [288], [349], [436], [437], [438].

In the context of surgery of the pituitary gland, the amount of liquor flow after the end of tumor surgery can be classified into different degrees:

- Grade 1: macroscopic preservation of the arachnoid, low CSF flow.
- Grade 2: defect of the arachnoid, moderate CSF flow.
- Grade 3: large defect of the arachnoid or dura defect, mostly in extended interventions.

The efforts of defect reconstruction increase with the grade [310]. In 80% of routinely performed interventions at the pituitary gland, no CSF flow is observed. In those cases, covering of the sella with absorbable material is sufficient for reconstruction [60], [427], [435], [439], [440]. 

### 3.7 Transplanum/transtuberculum interventions 

Transplanum-transtuberculum interventions are indicated in cases of midline suprasellar lesions such as for example craniopharyngiomas, Rathke or dermoid cysts or meningiomas of the tuberculum sellae. Beyond, it is indicated as “extended endoscopic endonasal access (EEEA)” (or “extended transplanum-transtuberculum approach (ETTA)”) (see above) in cases of dumbbell-shaped, only suprasellar, recurrent, or fibrotic pituitary adenomas. The same applies if a suprasellar part of an adenoma does not descend after transsellar relief. In single cases, also intraventricular tumors (papillomas) were resected [98], [119], [327], [385], [441], [442].

The initial surgical steps of transplanum-transtuberculum interventions are comparable to those of transsphenoid surgeries. Mostly, one middle turbinate is resected, the contralateral turbinate is lateralized and the dorsal nasal septum is removed. The posterior ethmoid is dissected on both sides, often the superior turbinates on both sides have to be resected in this context. All septa in the sphenoid sinus are abraded as well as the bone over the sella, the tuberculum, and at the sphenoid planum. It is also important to remove the bone from the medial opticocarotid recess. After wide sphenoidotomy, the access through the skull base is performed comparably anterior and superior. The superior intercavernous sinus is coagulated and the dura is transsected. In anterior direction, the opening reaches the base of the falciform ligament. Intradurally, the suprasellar cistern is exposed with the parasellar spaces; anatomically the difference is made between a suprachiasmatic, infrachiasmatic, retrosellar, and intraventricular area. In comparison to conventional neurosurgical accesses, the transnasal approach allows better visualization of the three last-mentioned areas [277], [385], [443]. In detail, different approaches are described locally for lesions of the different areas (e.g. prechiasmatic or subchiasmatic corridors). The pituitary gland can be lateralized with preservation of the superior pituitary artery in order to reach dorsal structures (retroinfundibular region, interpeduncular cistern) or mobilized in cranial direction [38], [93], [205]. Alternatively, the intervention is completed without displacement of the gland through a transclival access and performed by means of angled optics and appropriate instruments (“above and below approach”) [444].

The transplanum-transtuberculum intervention is easier and less dangerous for advanced sphenoid sinus pneumatization, larger sella, and thin bones in the area of the tuberculum. The access to suprachiasmatic areas is difficult when the chiasm is pre-fixed or displaced in anterior direction by a tumor [115], [445]. If retrosellar spaces have to be explored, a high positioned dorsum sellae or an extensive pneumatization of the clinoid process may have a negative effect. The quality of the intraoperative exposition of anatomical structures correlates with the distance of the carotid artery of both sides; in the area of the falciform ligament it amounts to about 15 (12–23) mm [165], [171], [385]. A suprasellar extension of the lesions to the level of the hypothalamus or the floor of the 3^rd^ ventricle are crucial and have a poorer prognosis. The anatomical relationship to neighboring structures and the proximal part of the anterior cerebral artery must be subjected to special analysis [112].

In about 70%, meningiomas of the tuberculum sellae grow into the canal of the optic nerve. As the tumor extensions within the canal are located mostly infero-medial, i.e. well exposable in the transnasal surgical corridor, bilateral decompression of the canals of the optic nerves is recommended, if needed [102]. 

The reconstruction after transplanum-transtuberculum interventions is a particular challenge. The secondary “dead space” in the suprasellar area after resection of a lesion is often filled with autologous or allogenic tissue. For stabilization, a second tissue layer or better a naso-septal flap is positioned. In order to avoid pressure on the optic nerve (primarily or secondarily after swelling of the transplants), however, “inlay grafts” are often consciously left apart even with the risk of a higher rate of postoperative CSF fistulas. The same aspects have to be discussed after opening of the floor of the 3^rd^ ventricle – in this context, an obstruction of CSF flow caused by adhesions of inlay transplantations must be avoided [226], [446]. 

### 3.8 Interventions at the cavernous sinus

Special interventions in the cavernous sinus are rarely performed because of the risk of postoperative cranial nerve deficits. In the focus is mostly an invasion of the sinus by pituitary adenomas, especially in cases of already existing cranial nerve palsies. In general, pituitary adenomas displace the internal carotid artery in lateral direction without tissue contact to the arterial wall. Based on imaging, this invasion of the sinus by adenomas can be described and quantified in stages (grade 0–4) according to Knosp et al. [447]. If an adenoma of the pituitary gland infiltrates the dura of the cavernous sinus, it can no longer be completely resected in many cases [239].

Meningiomas tend to early infiltrate the cranial nerves within the cavernous sinus – here an alternative radiotherapy must be discussed. 

Generally other tumors often locally compress the sinus and sometimes induce thrombosis so that there is no need to expect significant intraoperative bleeding at least at the beginning [214], [338], [448].

The carotid artery divides the medial compartment of the cavernous sinus from the lateral compartment with the cranial nerves III, IV, V_1_, V_2_, and VI. These different parts are reached via different corridors (Table 9 (Tab. 9)). In general, the intervention starts with ethmoidectomy with its accompanying interventions at the dorsal septum and the turbinates. If needed, transpterygoid expansion is also performed. An additional access via the contralateral side with posterior septectomy allows a better angle of preparation, if required. The anterior medial parts of the cavernous sinus can generally be better displayed than the postero-lateral and postero-medial (paraclival) parts. If necessary, the sella can be opened and the pituitary gland can be medialized; even the internal carotid artery can temporarily be displaced [214], [239], [313], [448], [449].

A realistic expectation of complete tumor resection (GTR, see above) is only possible in 50% of the tumors in the cavernous sinus. It is mostly achieved in cases of pituitary gland adenomas. If a transmaxillary-transpterygoid intervention for other tumor entities is performed, the rate decreases to about 17% [449].

After resection, the space is filled with fat tissue [448]. For further reconstruction, the naso-septal flap, fascia lata, or also alloplastic material (in combination) are recommended in literature [449]. 

### 3.9 Transpterygoid interventions

For ENT surgeons, transpterygoid interventions are of special interest as they are routinely performed for example to treat CSF fistulas in the lateral recess of the sphenoid sinus [450], [451], [452], [453].

By appropriately removing tissue, those interventions can reach many other, very different target structures in the pterygopalatine or infratemporal fossa, Meckel’s cave, the petrous apex, the lateral sphenoid sinus, and the cavernous sinus (Table 10 (Tab. 10), Table 11 (Tab. 11)) [30], [71]. The majority of interventions is done outside the dura [116], [254]. Preferably lesions are approached that grow rather in a displacing than in an infiltrating way [262].

Mostly, the lesions are located in the infratemporal fossa. Most often, the tumors continuously extend from neighboring structures into this space, e.g. from the paranasal sinuses, the nasopharynx, the parotid gland, or the middle cranial fossa (among others inverted papillomas, angiofibromas, chordomas, mucoceles, foci of fibrous dysplasia, squamous cell carcinomas, adenoid-cystic carcinomas, meningiomas, encephaloceles). Primary lesions in this area are very rare, in single cases, schwannomas, gliomas, neurofibromas, lipomas, lymphomas, sarcomas, adenoid-cystic carcinomas, and hemangio-pericytomas are found. Even more rarely, metastases are observed, e.g. of malignomas of the colon, thyroid gland, uterus, prostate, brain, or kidneys [101], [145], [232], [256], [267], [454], [455], [456], [457], [458].

The transpterygoid intervention starts with exposition of the sinonasal corridor: the middle (and in single cases the superior) turbinate is reduced. Then ethmoidectomy is performed with broad fenestration of the sphenoid sinus. The posterior septum is generously removed (in some cases up to the frontal plane at the anterior head of the middle turbinate or to the anterior edge of medial maxillectomy) – if needed, previously a naso-septal flap on the contralateral side is elevated and temporarily placed in the nasopharynx or the contralateral maxillary sinus. The next surgical step corresponds to the lateral extension of the intervention. Medial maxillectomy with resection of the inferior turbinate and closure (coagulation or clip) of the maxillary artery or the sphenopalatine artery is performed. The vessels are pushed in infero-lateral direction and the neural compartment of the pterygopalatine fossa is exposed. The preparation progresses in direction of the infratemporal fossa and the parapharyngeal space. In the focus hereby is the removal of the posterior wall of the maxillary sinus, vertical parts of the palatine and the pterygoid processes. Lateral and posterior of the lateral pterygoid process, the foramen ovale is reached – inevitable bleedings from the local venous plexus can be stopped by insertion of hemostyptic material. After the described exposition, the actual excision of the lesion is performed. The resection cavity is finally rinsed with clindamycin if needed; according to the location and extent of the lesion, appropriate reconstructive measures follow [146], [150], [250], [254], [267], [276], [289], [392], [426], [457], [459], [460].

In case of reduced interventions, it is suitable to avoid complete medial maxillectomy for example via a “prelacrimal approach” or leaving the anterior inferior concha in order to preserve nasal physiology; the resection of the nasal septum can be avoided by special “transseptal” instrumentation [251], [460], [461], [462], [463]. Modifications of the preparation were also described for preservation of the major palatine nerve and the Vidian nerve [255], [464].

In contrast, target structures lateral and posterior of the foramen ovale may require the transnasal extension of the access even after creation of a generous transnasal corridor in the sense of “endonasal surgery according to Denker” or a separate transoral approach (“2 ways/4 hands”). In other cases, the combination with a transethmoid, transorbital, or transsphenoid corridor is performed, in single cases also an additional (if needed minimalized, endoscopically controlled) transtemporal access from outside [69], [71], [116], [150], [206], [262], [276], [465], [466], [467], [468]. In this way, the instrumental range of action and working angles, the distance to the target structures, and the intraoperative view are optimized [145], [146], [147], [148], [149], [263], [289], [455], [466], [469], [470].

Anatomical landmarks are the Vidian nerve with its accompanying artery; it leads to the first “knee” of the carotid artery distal to the horizontal (petrosal) segment. Further landmarks are the pterygopalatine ganglion, the infraorbital nerve/maxillary nerve with the foramen rotundum, palatine nerve, maxillary artery / sphenopalatine artery / descending palatine artery / posterior nasal artery, Meckel’s cave, and the lateral cavernous sinus. The preparation can be performed to the petrous apex; with reference to the horizontal part of the internal carotid artery, this anatomical area may be subdivided into an infrapetrous and a suprapetrous part [39].

The transpterygoid corridor extends also to the petrous apex with Meckel’s cave of the trigeminal nerve. The antero-medial Meckel’s cave can be exposed by a nearly quadrangular bony corridor (“front door to Meckel’s cave”, “quadrangular space”). In medial and inferior direction it is limited by the internal carotid artery, lateral by the maxillary nerve (N. V_2_), and cranial by the abducens nerve (N. VI) within the cavernous sinus. Mostly the internal carotid artery is not completely exposed and the opening to the ganglion is only made up to the level of N. V_2_ in order to safely protect N. VI. For lesions in the lateral Meckel’s cave, the transoral approach is suggested in addition; posterior-lateral or superior parts of the cave should alternatively approached through a conventional neurosurgical access [101], [233], [254], [339], [471], [472], [473]. The endoscopic transnasal approach is suitable for lesions in the area of the inferior-anterior petrous apex; for superior-anterior parts, however, the conventional transcranial access is more appropriate [474]. 

Cholesterol cysts are the most frequent benign pathological lesions of the petrous apex. A permanent drainage to the sphenoid sinus is often the definitive treatment. The individual access is planned based on preoperative imaging. In case of medial cysts, often the transsphenoid or the transclival access are sufficient; the necessary manipulations along the individual access to the lesion are planned by use of imagined lines based on landmarks and secured with a navigation system. If the cysts or lesions are located in lateral direction, a more or less extended transpterygoid approach has to be chosen. As intraoperative landmarks, there are the opticocarotid recess, the foramen rotundum, the sulcus tubae auditivae and the Eustachian tube itself (medial and posterior of the foramen ovale) as well as the carotid artery with the foramen lacerum. Often, the second knee of the carotid artery must be exposed and displaced or the cyst must be identified superior to the petrosal course of the carotid artery. If required, a dissection of the Eustachian tube takes place with resection of about 1 cm of the distal cartilage. In some positive cases, the created drainage pathway of the cyst could be optimized by applying balloon dilatation. Afterwards, a silicone drainage can be inserted for 3–4 months or a small pedicled flap is applied. The recurrence rates according to the literature are given with optimistic 5–12% [26], [262], [407], [436], [475], [476], [477], [478], [479], [480].

In direction of the nasopharynx, interventions of the superior parapharyngeal space may turn into “nasopharyngeal endoscopic resection” (“nasopharyngectomy”). In selected cases, it may also be applied for treatment of malignomas (Table 12 (Tab. 12)). Generally, the carotid artery may be tracked, exposed and isolated from the parapharyngeal to the cavernous part. An important landmark of preparation is the Eustachian tube (e.g. at the transition of the bony to the cartilaginous course), which may often be resected in these cases [126], [253], [273], [289], [481], [482].

In the majority of the cases, an excision of the lesion during transpterygoid surgeries is only incomplete [254]. In a heterogeneous patient population treated with an often multimodal therapeutic concept, a local control rate was mentioned with 65% [142]. There are also reports about for example a large recurrent epidermoid cyst of the middle cranial fossa and the infratemporal fossa that could by drained only by means of a silicone drainage tube in the maxillary sinus [483]. After extended removal of the pterygoid muscles, masticatory dysfunction must be expected. After more extensive manipulations at the tube, otological sequelae may occur [146], [455].

### 3.10 Transclival interventions

Transclival interventions aim at midline lesions of the clivus with limited lateral extension, in single cases also at foci at or even in the ventral brain stem. Examples of the first group are chordomas, chondrosarcomas, dermoids, epidermoids, cysts, and meningiomas; single cases of the second group are prepontine neuroenteric cysts, chordomas, cavernomas, or also gliomas.

Regarding target structures at the inferior clivus, the specific merit of transnasal surgery is a missing contamination by the oral flora and a more rapid postoperative restart of oral nutrition in comparison to transoral interventions, without the risk of palatine dehiscence, dysphagia, or regurgitation [384], [484].

Generally, a “panclival” approach allows exposing a rectangular dura surface in front of the brain stem from the tuberculum sellae to the arch of the atlas. Laterally exposure is limited in the petroclival region by the internal carotid artery, the abducens nerve as well as the hypoglossal canal [72], [84], [485].

Depending on the segment of the clivus with its neighboring structures aimed at, the interventions mostly start with a transsphenoid access, i.e. posterior ethmoidectomy and exposition as well as wide opening of both sphenoid sinuses. An extended pneumatization of the “clival type” facilitates manipulations. The pituitary gland is mostly exposed and sometimes compressed or displaced by means of different techniques (extra- or intradural transposition, interdural hemitransposition). This procedure allows abrading the posterior clinoid processes. Parts of the vomer can be gained from posterior septectomy and stored for reconstruction. Beside the sella, first local landmarks are the protrusion of the internal carotid artery, the optic nerve, and the transition of the floor of the sphenoid sinus to the superior clivus. Starting at the sella, the bone of the posterior wall of the sphenoid sinus and the clivus are drilled down exposing the carotid artery. The inferior two third of the clivus are approached via a U-shaped, caudally pedicled flap of the nasopharyngeal mucosa together with the pharyngeal fascia and the prevertebral muscles. Afterwards the bone is drilled down which may cause bleedings in the venous plexus that have to be addressed. If the necessity occurs to perform intradural preparation, a vertical incision of the dura in the median plane is recommended in order to protect the laterally located abducens nerve in Dorello’s canal. Caudally, the incision can be extended in form of a reversed Y. After cauterization of parts of the intercavernous sinus and opening of the dura, the superior basilar artery, the vertebral arteries, and the posterior and superior cerebellar arteries as well as the pons cerebri may be identified [38], [62], [165], [235], [237], [472], [486], [487].

After removal of the inferior third of the clivus, the access can be extended optionally in lateral direction. By means of an additional transpterygoid corridor, a ventro-medial condylectomy to the canal of the hypoglossal nerve can be performed (“far medial approach”, in analogy to the transcranial “far lateral approach”). In this way, lesions located ventral of the vertebral artery and ventromedial of the hypoglossal nerve can be exposed. Control of up to 70% of the foramen magnum is reported to become possible [64], [99], [103], [488]. Further resections of or at the condyle may lead to secondary cranio-cervical instability. Tumor extensions behind the petrous apex require surgical exposition and transposition of the petroclival internal carotid artery; a technical variation with transposition of the Eustachian tube was described [489], [490]. Alternatively, the transclival approach may be expanded by use of angular optics in combination with flexible or special controllable instruments. In other cases, a combination with transcranial and sometimes also transoral/transpalatine interventions is performed [64], [99], [124], [164], [301], [330].

All transclival interventions require careful multilayer reconstruction of the dura defects applying pedicled flaps (see below) [491], [492].

Based on the several studies, the rate of complete resection (GTR, see above) in case of malignomas amounts to about 55% [493].

### 3.11 Interventions at the cranio-vertebral transition (transodontoid interventions)

Caudal of the transclival access, there is space for mostly extradural interventions at the cranio-vertebral transition (transodontoid interventions). They are performed via bilateral transnasal corridors alongside the palate. The dorsal vomer is resected, the soft palate is transposed in caudal direction by traction tubes. A caudally pedicled mucosal flap of the posterior wall of the pharynx is created and placed in direction of the oropharynx. Alternatively, a median mucosal incision is performed pushing the muscles aside. The inferior parts of the clivus are removed if necessary. A horizontal line between the superior edges of the Eustachian tube of both sides crosses the anterior edge of the foramen magnum and may serve as auxiliary line. Following, the atlanto-occipital membrane, the longus capitis muscle, the longus colli muscle, and anterior parts of C1 and C2 are exposed. The anterior arch of the atlas can be resected. If necessary, also the dens of the axis is removed. This approach serves for resection of rheumatoid lesions or other bone diseases, tumors, cysts, or metastases; other indications are cranio-cervical malformations, basilar impressions, or post-traumatic dislocations of the dens. The intervention is performed with monitoring of the cranial nerves and under constant control of the brain stem potentials (BERA). If the anterior arch of C1 can be preserved, the endonasal intervention at the dens leads to a relatively low destabilization of the cervical spine. In case of C1-2 resection, the patients generally undergo successively (i.e. primarily) or simultaneously (in the same session) posterior, occipito-cervical fusion with fixation [26], [104], [138], [292], [296], [337], [494], [495], [496], [497].

Different auxiliary lines with regard to sagittal median CT sections should mark the inferior limit of the transnasal endoscopic access to the cranio-cervical transition:

A. “palatine line” alongside the hard palate, i.e. from the anterior nasal spine to the posterior nasal spine.

B. “naso-palatine line” from the rhinion to the posterior nasal spine [26], [140], [498], [499].

C. “naso-axial line” from the middle of the distance between the “anterior nasal spine – rhinion” to the posterior nasal spine [141].

The height of the cranio-vertebral transition in relation to the palatine line is subjected to individual variations [500]. In the axial level, the hard palate should be at the level of the foramen magnum [162]. The last-mentioned naxo-axial line describes best the endonasal corridor, it generally ends in the area of the anterior surface of C2 [139], [141], [500]. The endonasal intervention often seems more challenging in comparison to traditional transoral surgeries; the corridor is described as being narrow and deep [495], [501]. However, the following advantages are mentioned: surgical splitting of the palate or oropharyngeal incisions can be avoided. The view on the foramen magnum is comparably good, the odontoid process is identified early. The endoscope allows good control of the drilling and, if required, protection of the anterior arch of C1. Specific spatial or strategical advantages are observed in macroglossia, micrognathia, or a high position of the odontoid process. The subsequent postoperative morbidity is less severe with regard to swallowing (velo-pharyngeal incompetence). The physiology of the upper airways is better preserved, the rate of meningitis is lower, and reconvalescence occurs more rapidly [26], [140], [495], [502]. The different accesses may be combined if necessary [61]. An optimized respective procedure is based on an altering endoscopic control via the transnasal or transoral corridor with instrumentation via both nasal cavities [292].

## 4 Special reconstructive procedures

In historical patient populations an unacceptably high rate of secondary CSF fistulas (20–50%) was observed after transnasal interventions at the skull base [380], [503], [504]. Thus, special reconstructive procedures in rhino-neurosurgical interventions are considered as being indispensable when the dura defects are large, a high CSF flow must be expected, and when no circumferential bony edges of the skull base defects are at disposition for fixation of free transplantations [505], [506]. Patients with significant adipositas (increased ventricular pressure), Cushing’s disease (delayed wound healing), and after previous surgeries or (chemo-)radiotherapy have a higher risk to develop liquor fistulas. Other factors revealing a higher risk for complications following skull base reconstruction are: a patient’s age over 60 years, cardiological or pulmonary comorbidity, diabetes, existing intracranial infection [505], [507].

Minor interventions at the pituitary gland often have a special status. A particular grading of the defects was described [508]. If the sellar diaphragm was preserved, if the suprasellar tumor growth or a prolapse of the parasellar cistern was missing, and if intraoperative bleeding from the cavernous sinus did not occur, the challenge of reconstruction is minimal: sometimes it is possible to do without special reconstructive measure, sometimes only some fat tissue is inserted [379], [403], [509].

Generally the size, position, and origin of the dura defect as well as the type and the material of the closure are less important for the success of the measure than an appropriate surgical technique [492], [510], [511], [512], [513]. Those principles are known from surgery of the paranasal sinuses [11], [399]. Persistence or recurrent fistula can develop based on intracranial hypertension. Suspected cases have to be examined by lumbar puncture with pressure measuring “under physiological conditions” about 3–5 days after closure of the fistula or 24 hours after the postoperative removal of the lumbar drainage [512], [513], [514].

Algorithms of reconstruction were described for defects of different locations [380], [515], [516]. Mostly a multilayer closure with “inlay” and “onlay” transplantation is performed [517]. Often it is difficult to insert inlay transplants in the sphenoid sinus or at the clivus because of missing stable bony rims [514]. Near delicate neurovascular structures, preferably overlay transplantations should be applied in order to minimize manipulations close to the structures [440], [518]. Special care must be taken that intradural displacement of transplants is avoided [508]. For multilayer treatment of CSF leaks with high flow-rate (“high flow leaks”), pedicled flaps are required [492].

### 4.1 Materials and procedures

- Autologous tissue:
  - Fat to minimize surgical cavities (as inlay) before covering e.g. with pedicled flaps, to fill parts of the sphenoid sinus or to cover small residual defects (onlay) [383], [118], [512], [513], [517], [519], [520].
    In case of intracranial insertion of fat tissue, a compression of the chiasm or the optic nerve must be avoided [93], [521], [522]. If the 3^rd^ ventricle was widely opened, fat should be used reluctantly in order to prevent migration into the ventricle with local adhesion (resulting in an iatrogenic hydrocephalus) [92], [516]. Special techniques to avoid a displacement of the fatty tissue were described [523]. The insertion of fat can make it difficult to perform postoperative imaging with the aim of identifying residual tumor tissue [119]. An absorption of ≥50% of the fat tissue must be expected [524].
  - Autologous fascia (mostly fascia lata; alternatively fascia of the temporalis muscle or the abdominal rectus muscle) as inlay or onlay transplantation [383], [108], [519], [522], [525]. The thickness of a fascial transplant is reduced postoperatively by ≥50% [524].
  - Autologous bone, e.g. taken from the vomer or cartilage of the nasal septum [92], [93], [118], [526]. Bigger autologous bone pieces, however, tend to cause healing problems and extrusion after irradiation [2], [491].
  - Autologous nasal mucosa or muscle [315]

- Allogeneic tissue:
  - Allogenic fascia lata, pericardium, and if necessary dura [353], [517], [527], [528], [529].
  - Allogenic acellular dermis (AlloDerm^®^; acellular dermal allograft) [75], [303], [530]. The dermis is internationally applied in ‘off-label use’ as onlay transplant [55], [531]. If needed, it can be fixed with U clips at the dura defect edges (see below) [517].

- Allogenic or xenogenic collagen preparations as inlay or onlay transplants [383], [519], [520]. In this context the different biomechanical and biological properties of the individual materials must be taken into account [532]. For certain interventions (e.g. transsphenoid surgeries), xenogenic transplants are not recommended [283].
  - Xenogenic collagen membrane made of bovine (Achilles) tendon (DuraGen^®^) [2], [75], [93], [205], [307], [504], [531]. The material is used as an inlay transplant; it is not suitable as an onlay transplant. Furthermore it has to be combined with other modalities of reconstruction [168], [517], [531].
  - Xenogenic collagen membrane made of bovine pericardium (DuraGuard^®^; Tutopatch^®^) [82], [93], [95], [302].
  - Xenogenic equine or bovine collagen fleece (TissuFleece E^®^) [403], if needed in combination with allogeneic fibrinogen and thrombin (TachoSil^®^, Avitene^®^) [55], [292].

- Alloplastic material is often controversially discussed, an increased rate of infections with sequestration is reported [36], [531]:
  - Fine fibrillary microporous fleece of polyester urethane (Neuro-Patch^®^) [417].
  - Porous synthetic implants (e.g. Medpor^®^/Porex^®^) [82], [92], [97], [118], [380]. The implants are suspected to bear a significant infection risk [507].
  - Synthetic mixtures of polylactid and polyglycolic acid (LactoSorb^®^) [115], [309], [527], [528].
  - Hydroxyapatite cement [280]. Frequent local infections were reported [312], [507].
  - Titanium plates, miniplates [93], [192], [508], [533]. With regard to healing disturbances, the use of titanium is not recommended [491].
  - Highly viscous polymethyl-methacrylate [534].

- Additional tools that are applied are:
  - Fibrin glue or collagen fleece, also for filling “dead remote spaces” [403], [535]. For direct and dosed application of the glue, it is recommended to use long flexible cannula tips that are inserted and advanced via curved suction devices [536]. Some authors, however, explicitly refuse such sealing [537], [538].
  - Synthetical polymerized hydrogel of 2 components as dura sealing mass (DuraSeal^®^) [82], [93], [118], [302], [539]. The material is applied from outside on the reconstructed area. If it gets for example under a mucosal flap, healing may be disturbed. The material takes 4–8 weeks for absorption [41], [120].
  - Oxidized cellulose, mostly for coverage of transplantations as second or third layer [303], [307], [504].
  - Gelatin [93], [303], [504]. Gelatin, soaked for example in gentamicin, is used to cover transplantations [446].
  - Nitinol U clips (e.g. Medtronic U-clips^®^). It is an ‘off-label use’ of clips usually applied in cardiovascular surgery that tighten themselves with a “memory function” [55], [168], [504], [540]. Titanium clips that are usually applied in vascular surgery work in a similar way when used for direct closure of dura defects [541]. Alternatively, transplantations can also be fixed with sutures [55]. In single cases, also a direct dura suture may be successful [542].
  - Silicone sheets to cover transplantations [403], [417].

Frequently applied for a multilayer treatment of dura defects is for example the so-called “triple F” reconstruction (“fat + fascia + flap”) [295], [543]. Those materials are finally covered with fibrin glue or synthetic hydrogel. Selectively, a reinforcement with oxidized cellulose is performed, if necessary. Another layer may be gelatin. Intraoperative Valsalva manoeuver may help to control the result [331], [353], [544].

Finally, the nose is packed inside with sponge tamponades or closed with an endoscopically inserted balloon [74], [383], [446], [518], [520], [525], [531].

For special cases or as alternative, modifications are described. At the anterior skull base, often a single or double inlay of fascia lata with an additional fascial overlay is sufficient [544]. A combined closure of dura defects with soft tissue and hard material analogous to gaskets (“gasket seal”) was positively considered in the literature [97], [302], [522], [533], [545]. However, the precondition for application of this technique are planar defects with stable bony rims and so it cannot be applied for example for defects in the area of the pituitary gland [546], [547]. Other particular closure techniques have been described as “bilayer button” [548], “bath plug” [549], or “champagne cork technique” [550].

### 4.2 Defect coverage with autologous regional tissue

1. Free autologous mucosal flaps for smaller defects. The flaps have to be prepared clearly larger (+25%) than primarily measured in order to calculate inevitable postoperative shrinking [551].
2. Pedicled local flaps:
3.
  1. **Flaps with pedicle in the area of the sphenopalatine artery**
    *1. Posterior pedicled naso-septal flap.* The whole septal mucosa of one side is circumcised in the cranial, caudal, and anterior circumference. Dorsal, a pedicle is outlined over the inferior anterior wall of the sphenoid sins containing one branch, sometimes 2 branches, of the sphenopalatine artery (see below). This flap is the mostly used one having a surface of about 20 cm². By special extension including the mucosa from the nasal floor and the inferior nasal meatus, the surface may be increased to about 27 cm² [383], [552], [503], [553], [554].
    *2. Posterior pedicled flap of the inferior turbinate.* With an electronic needle, sagittal incisions near the superior attachment of the inferior turbinate and in the inferior nasal meatus are performed. At the tip of the turbinate, both incisions meet. Then the mucosa is carefully taken from the turbinate bone and the inferior lateral nasal wall. Some authors remove the turbinate bone, others protect it. A relatively narrow flap of 2×5 cm is created. Preferably, defects in the sphenoid sinus and at the clivus, if required also in the area of the posterior part of the frontal skull base, may be covered [491], [503], [555], [556]. The inferior turbinate flap can be elevated bilaterally [551].
    *3. Posterior pedicled flap of the middle turbinate.* Via a vertical incision at the anterior edge of the middle turbinate, the mucosa is taken subperiostally from the bone of the medial and lateral surface as well as the inferior free edge. Parts of the bone are removed. At the level of the “axilla”, a horizontal incision of the vertical turbinate lamella is performed in direction of the superior nasal meatus. After detaching from the bone, the mucosal flap may be opened “like a book” and pursued with its pedicle in direction of the foramen spheno-palatinum. A relatively short flap of about 3×4 cm results which can be used for defects in the area of the sella, the sphenoid planum, and at the posterior roof of the ethmoid sinus [503], [551], [557], [558]. One negative aspect is the often insufficient quality of the mucosa and a natural traction via the pedicle away from the skull base [531].
  2. **Flaps with anterior vascular pedicle **[559]:
    *1. Anterior pedicled inferior turbinate flap.* This flap has its pedicle in the area of the peripheral plexus of the ethmoid artery in front of the agger nasi. The result is an anterior-superior pedicled, relatively narrow flap measuring about 2×5 cm – if needed, it must be combined with other flaps. The flap is used for defects e.g. in the area of the posterior wall of the frontal sinus or the lamina cribrosa with adjacent roof of the ethmoid sinus. The uncovered bone of the inferior concha is subject to secondary re-epithelization [560], [561].
    *2. Anterior pedicled mucosal flap of the inferior lateral nasal wall and the nasal floor.* The flap has a similar pedicle as the anterior turbinate flap. Together with the whole mucosa of the inferior turbinate (if necessary including the fontanel of the middle meatus), however, also the mucosa of the inferior meatus (leaving out the ostium of the lacrimal duct) and the nasal floor is elevated. The flap should be sufficient to cover large parts of the anterior ethmoid roof [515], [562].
    *3. Bipedicled flap of the anterior septal mucosa.* With a vascular pedicle via the superior labial artery and also the arterial supply in the incisive canals, a horizontal, anterior pedicled mucosal flap is created in the area of the anterior-superior septal mucosa. A flap of 4×2.5 cm may be prepared. It can be moved of about 90° in cranial direction and used for covering the posterior wall of the frontal sinus at about half of its height [563]. A similar flap may be created as a transpositional flap from the anterior-superior septal mucosa that can be moved to the contralateral side of the skull base [491].
  3. **Regionally pedicled tissue transfer (less frequently used):**
    *1. Transglabella-pericranium flap.* Via two incisions (2 cm, 1 cm) in the hair bearing skin near the vertex a big pericranium flap is circumcised with a stalk of 3 cm in width in the area of the supratrochlear or supraorbital artery. Following another skin incision at the nasion, a 4×15 mm horizontal slit can be drilled in the bone of the nasal root. Via this access, the flap is transposed into the inner nose after having been applied a frontal sinus drainage type III [515], [564], [565]. The individual dimension of the flap can be calculated preoperatively, e.g. before covering a defect in the area of the sphenoid sinus, based on data of the CT scan. However, for defect coverage in the area of the posterior skull base and the clivus, this flap is not suitable [505], [566].
    *2. Transpterygoid temporo-parietal fascial flap.* Via a hemicoronary skin incision, a bigger caudally pedicled flap of the temporal fascia is circumcised. After appropriate preparation (antrostomy with partial removal of the lateral and posterior walls of the maxillary sinus, if needed resection of parts of the pterygoid processes with shifting of the muscles), the fascial flap can be advanced from lateral into the internal nose (possibly using the instruments of percutaneous tracheostomy). The flap has a relatively large surface and is suitable for irradiated patients [515], [564], [567], [568]. It is less appropriate for defects of the anterior skull base [505].
    *3. Palatine mucosal flap.* The mucosa at the hard palate and at the alveolar process is circumcised intraorally with a pedicle in the area of the descending palatine artery. Intranasally, the dorsal part of the mucosa is elevated at the nasal floor and temporarily transposed in medial direction. The inferior turbinate is detached anterior at the base, but it remains dorsally pedicled. After extensive removal of the bone at the foramen and alongside the greater palatine canal with mobilization of its arterial stalk, the palatine mucosal flap is transposed from oral in nasal direction. The inevitable regional palatine defect is closed by folding back the nasal mucosal part on the nasal floor with replacing the turbinate. Intraorally, the donor defect is closed with acellular dermis, if needed (see above) and covered with a dental plate for 2 weeks [515], [564], [569], [570]. The literature on clinical experience is limited, the donor defect is functionally relevant – so that the flap is considered to be the ultima ratio [505].
    *4. Pedicled flap of the buccinator muscle.* Protecting Stenon’s duct, the buccinator muscle is circumcised including a cranial pedicle of the facial artery – the supply of the flap occurs retrograde via the angular artery. The local oral mucosa is only taken together with the flap if particularly needed. After medial maxillectomy and additional osteotomies, the flap is advanced in the nasal cavity and used for reconstruction of defects of the anterior skull base [515], [571]. Regarding the application of the flap, the same aspects apply as for the palatine mucosal flap [505].
    *5. Occipital galea-pericranium flap.* Via external incision, a galea-pericranium flap measuring maximally 11×4 cm can be gained from the occiput. The pedicle consists of the occipital artery with accompanying vein. The flap and stalk are prepared and elevated. After performing endonasal medial maxillectomy, removal of the medial posterior wall of the maxillary sinus, and abrasion of parts of the pterygoid processes, the flap may be advanced alongside the infero-medial medial pterygoid muscle via a prepared and widened tissue canal into the nose. This flap is appropriate for defects of the posterior frontal skull base and its transition to the middle cranial fossa [515], [572], [573].
  4. **Free pedicled flaps (less frequently applied):**
    *1. Free pedicled forearm flap.* A hybrid approach is performed, i.e. a combination of endonasal endoscopic surgery with a transoral access. After performing modified medial maxillectomy, the forearm flap is inserted for defect coverage and the vascular pedicle is conducted via the maxillary sinus and the buccal soft parts to the facial artery and vein. The preparation of the donor vessels and the vascular anastomosis are performed via a small submandibular skin incision [515], [574].

### 4.3 Additional information on the naso-septal flap

By the time the pedicled naso-septal flap has become an elementary rhino-neurosurgical technique. Not least because of its application, the rate of postoperative CSF fistulas after rhino-neurosurgical interventions could be reduced to partly less than 5% [554].

The flap is especially suitable for defects in the area of the dorsal skull base, the sphenoid sinus or the clivus. The anterior defect closure near the frontal sinus access is critical; in case of resections of the skull base from one orbita to the other, the width of the flap must be increased by including mucosa of the nasal floor. Defects of extended combined interventions (several modules) are not always appropriate for this flap [69], [520], [553], [557], [558], [575], [576]. In about 10% of the patients, there is generally no possibility to create the flap [515]. In those cases, the pedicle of the septal mucosa had been destroyed at the occasion of previous surgeries or foci of a disease (e.g. tumor infiltration, septal perforation). An intact pedicle can be proven by Doppler probe, if required [577]. In the individual case, previous transcranial surgery is no contraindication [578]. If a transpterygoid intervention is planned, the flap should be elevated preferably on the contralateral side in usual technique or in case of need on the ipsilateral side in a modified manner [579]. A possible septal spur with atrophic and vulnerable mucosa must be taken into special consideration of the flap design [107], [503], [505], [525], [533], [580], [581].

Naso-septal flaps can generally be created also in children [136], [504], [554], [582], although this procedure should be performed reluctantly because of possibly damaged growth centers in the nasal septum [132]. In children younger than 10 years, the surface of the flap is critically small [504], [505], [583].

The pedicle consists of mostly 2 (>70%) branches of the posterior nasal artery or posterior septal artery (originating from the sphenopalatine artery). They run over the inferior anterior wall of the sphenoid sinus in a distance of 5 (to 9) mm to the sphenoid ostium and are incorporated in the pedicle of the flap [584].

The naso-septal flap has to be elevated at the beginning of surgery and temporarily displaced into the ipsilateral maxillary sinus or nasopharynx. Only in this way, the flap and the vascular stalk can be left off the surgical field and protected during further preparation. This timeline explains why the dimension of the later dura defect and the size of the flap have to be obligatorily calculated beforehand [525], [581].

If, unexpectedly, the flap is not needed and no posterior septectomy has been performed, it can be re-sutured in situ and re-elevated perhaps at a later time [446], [539], [585]. If the need of a flap cannot be foreseen in case of transsphenoid surgeries, alternatively a modification of the surgical technique (**“nasoseptal rescue flap”**) can avoid the prophylactic complete elevation of the nasoseptal flap. The incision is limited to parts of the pedicle and the mucosa is initially mobilized only regionally out of the direct surgical corridor. With the same objective, a 15×5 mm measuring mucosal area can be excised directly medially of the sphenoid ostium. If the flap is not needed, the mucosa is re-placed; in other cases, the incisions are completed after identifying the situation with its needs [584], [586], [587], [588], [589], [590]. When the flap at the nasal septum is circumcised, the superior 10 (to 25) mm of the mucosa with the local olfactory region (“septal olfactory strip”) must be protected. Only in front of the level of the anterior medial turbinate, the incision can be conducted up to directly under the ridge of the nose. The use of electrosurgery for this posterior-superior incision (and often generally) is not recommended. In this way, the olfactory mucosa should be protected, shrinking is avoided, the vitality of the edge of the flap is preserved, and epithelization is facilitated [518], [591], [592], [593], [594]. Despite all this, in single cases (4%) a postoperative clinically apparent hyposmia must be expected [383], [575], [581], [595], [596], [597].

Attaching the elevated flap in the area of the defect, attention must be paid that the pedicle remains in contact with vital tissue in order to avoid desiccation with subsequent retraction [554], [575]. A general postoperative shrinking of the flap of about 20% is predicted in the literature [64], however, other authors deny this statement [575].

For fixation of the flap in the wound, often a 12-14 French Foley catheter is inserted for 3–7 days. In single cases, pyramid-shaped balloons known from midfacial traumatology (for stabilization of the orbital floor), Fogarty catheters, or often even simple sponge tamponades are used. The bare cartilage and bone at the septum are covered by silicone sheaths for about 10 days to 4 weeks [55], [122], [205], [295], [383], [520], [525], [594], [598], [599].

According to the literature, a nasoseptal flap can be elevated simultaneously on both sides [82], [383], [519], [575], [582], [595], [600]. In single cases this is also possible for children, as stated in the literature [582]. Other authors advise against bilateral elevation [554]. To protect the nasal physiology, a modified surgical technique as so-called Janus flap was described for bilateral interventions [533], [601].

The donor defect at the nasal septum is often subject to spontaneous re-epithelization after application of the nasoseptal flap. The result may be crusting for 3–6 months, sometimes even septal perforations [520], [525], [575], [576], [581].

The unfavorable results for nasal physiology can be reduced by a **reverse flap** from the contralateral septal mucosa [602], [603], [604]. The same is true for free transplantation of nasal mucosa on the exposed septum [605].

The success rate of defect covering with nasoseptal flaps amounts to 85–95% [383], [519], [520], [525]. Defects with a surface of more than 2 cm² or with high liquor flow rate (i.e. after intraoperative opening of cisterns or ventricles) and defects in children or patients after radiotherapy are more likely to cause problems [520], [606]. If necrosis of the flap becomes apparent, the immediate re-surgery is performed with an alternative technique of reconstruction. In single cases, the development of mucoceles after insertion of nasoseptal flaps for example into the lateral sphenoid sinus was reported – other authors do not share this experience [491], [533], [607], [608], [609]. Even after defect closure at the skull base applying simple free autologous mucosa transplants, mucoceles are observed [610]. In postoperative coronary or sagittal MRI sections, the nasoseptal flap is mostly displayed as a C-shaped structure. Its thickness is 2–8 mm, and with intact vitality it is isointense to the brain especially in the T2 mode. The adjacent mucosa has a different signal pattern [519], [595]. After application of contrast material, a vital flap is clearly marked – a divergent signal intensity and also a moderately altered position of the flap, however, does not indicate a higher risk of insufficiency of the flap with the exception of larger dura defects with a high flow leak [519], [595], [611]. With further healing progress, the thickness of the flap decreases of 20–30% [524].

## 5 Surgical outcome after typical interventions, quality of life (QOL)

A complex summary of the results of surgical therapy of all usual tumor entities was presented by Lund et al. as **“European Position Paper on Endoscopic Management of Tumours of the Nose, Paranasal Sinuses, and Skull Base”** in 2010 [52].

In the following, only additional information is given. It has to be noted that most of the reports on surgical results refer to very heterogeneous patient populations, differential indications, therapeutic approaches, and modalities of data collection. So the literature can only be assessed in a limited way, e.g. according to sound principles of evidence-based medicine [57], [94], [131], [612], [613].

Generally patients recover very well after rhino-neurosurgical interventions. Preoperative deficits of vision improve in 50–90% [117], [283]. Ratings on the general quality of life are reduced postoperatively and recover after one year at the latest to comparable values as before surgery. A tendency to no-change is observed in impaired olfaction and tasting as well as nasal secretion or crusting. For those particular problems the usual rhinological questionnaires are only suitable in a limited way [614]. Besides a questionnaire entitled “ASBQ – anterior skull base questionnaire” on the quality of life after oncological subcranial interventions [615] and the “Midface Dysfunction Score” [616], meanwhile also a questionnaire entitled “Skull Base Inventory – SBI” [617] as well as an “Anterior Skull Base Nasal Inventory – ASK Nasal-12” have been presented [618], [619]. Special questionnaires on the endocrinological function are used separately [617].

The postoperative health condition does not correlate with the tumor stage. Older patients (>60 years) recover less well from an intervention; the same is true for patients undergoing postoperative radiation. In some investigations, the condition of female patients is slightly less favorable [52], [599], [620], [621], [622], [623], [624], [625].

Careful indication and selection of patients lead to oncologic results of endoscopic interventions that are comparable to those of transcranial or craniofacial resections [6], [57], [626], [627], [628], [629]. In case of malignomas, the interventions are terminated with about 17% positive or very narrow histological margins – also in this context there is no difference to comparable conventional surgeries [630].

Endoscopic interventions are most frequently performed in tumors of limited extension [629]. The quality of life of children and adults after transnasal interventions is better than after conventional surgeries. Sino-nasal complaints, however, are often considered as being similar [326], [613], [631], [632].

### 5.1 Pituitary tumors

According to reviews of the literature on endoscopic surgery, the rate of complete resection (GTR) of tumors of the pituitary gland amounts to 70–80% [30], [60], [131], [427], [633]. For revision surgeries, the rate is about 2/3 – if the previous surgery was performed with the microscope, the rate is slightly higher [634]. Even for very large tumors (“giant adenomas”: ø ≥3 cm, volume ≥10 cm³) a rate of 24% can be achieved [635]. 6–10% of the adenomas grow into the cavernous sinus and make an expansion of the surgical intervention inevitable – the success rate (GTR) amounts then to 2/3 of the cases [59], [636].

The endonasal approach is comparably less traumatizing. Subsequent nasal complications occur more rarely (postoperative bleeding, septal perforation, saddle nose, disturbances of sensitivity of the upper lip or the maxillary teeth, anosmia). The postoperative time of reconvalescence is shorter [111]. A postoperative compensation of a hormonal imbalance must be expected in slightly more than 80% [633]. Preexisting visual impairment improve in more than 50% [634].

### 5.2 Encephaloceles, meningoceles, CSF fistulas

Endonasal surgery is meanwhile an established standard procedure for treatment of post-traumatic, iatrogenous, neoplastic, or congenital skull base defects or spontaneous fistulas with and without increased cranial pressure. According to their location, meningo-encephaloceles are classified in transethmoid, spheno-ethmoid, spheno-orbital, spheno-maxillary, and transsphenoid lesions. Within the sphenoid sinus, the difference is made between medial-perisellar and lateral types [26], [329], [637].

Lateral meningo-encephaloceles of the sphenoid sinus are correlated with a persisting Sternberg’s canal [638]. The treatment of those defects requires sometimes a modified transpterygoid approach (“EPSEA – ethmoid-pterygoid-sphenoidal endoscopic approach”) [637], [639].

There is a high heterogeneity of lesions, local anatomy, constitutional accompanying factors, and subsequently also surgical details.

Generally, the transnasal surgical treatment is also possible in early childhood [136], [137], [582]. The tissue of the cele is either preserved and gently pushed back or electro-coagulated and resected. Alternatively radiofrequency ablation can be performed. The defect is covered in a multilayer technique, if required, applying e.g. the nasoseptal flap [312], [329], [640], [641], [642].

A literature review reveals that the endoscopic endonasal surgery for the treatment of skull base defects is as safe and effective as traditional interventions. Defects in the area of the posterior wall of the frontal sinus are suitable for endonasal access only in a limited number of cases [643].

### 5.3 Meningiomas

The transnasal approach has several defined advantages for the treatment of meningiomas: the exposition of the tumor does not require a retraction of the brain, acting via the surgical corridor the tumor is early devascularized and the hyperostotic bone in the area of the tumor base can often be resected without significant efforts [283], [644],[645], [646], [647].

In ¼ to ¾ of the cases, meningiomas of the tuberculum sellae extend into the canal of the optic nerve; the optic nerves and the chiasm are displaced in cranial and lateral direction. Transnasal surgery allows early exposition and relief of the optic nerve with the functionally important subchiasmatic vessels [37], [63], [93], [97], [100], [119], [353], [648], [649], [650].

Meningiomas of the anterior skull base often have a supplying vessel from the distal internal carotid artery near the opticocarotid recess; often the ethmoid vessels are secondarily dilated [37], [183], [651]. In up to 15% of the cases, the tumors extend from the olfactory fossa into the ethmoid sinus. Those lobes are inevitably removed via the transnasal corridor [320], [647]. In case of smaller tumors of the rima olfactoria it may be possible to preserve the olfactory nerves with the olfactory mucosa on one side [353].

In contrast, in other cases it is mentioned as adverse that the cranial nerves and vessels may only present relatively late during the intervention in individual cases. The extracapsular preparation in direction of the brain tissue can be difficult when the pia mater is infiltrated. As an indicating sign of this fact, a hyperintensity in the T2 weighted MRI in the area of the tumor periphery is considered [644], [645]. 

In general, surgery strives for complete resection of the tumor while excluding particular risks. According to Simpson, the resection can be classified as follows [652]:

- Grade I: macroscopically complete tumor resection with excision of the dura adhesion and all abnormal local bone tissue.
- Grade II: macroscopically complete excision of the tumor with its visible processes and coagulation of the dura adhesions.
- Grade III: macroscopically complete resection of the intradural tumor without resection or coagulation of the dura adhesion or extradural processes (e.g. invasion of a sinus or hyperostotic bone).
- Grade IV: partial tumor resection.
- Grade V: decompression or biopsy of the tumor only.

In the literature, additionally grade 0 is mentioned (excision of a 2 cm strip of the dura around the tumor) [644], [645]. The classification according to Simpson is problematic as the recurrence-free postoperative interval does not correlate with grades I–III [646]. Grades III and IV lead to recurrence rates of 30–40% [52].

Only rarely, meningiomas with parasellar extension and petroclival processes are completely removed [183]. The predominant aim of therapy in these cases is the reduction of the tumor with improvement of the visual symptoms [26].

Generally, a tumor diameter of more than 35 mm in case of sellar meningiomas is considered as being critical, as well as a lateral extension over the supraclinoid carotid artery and close contact of the tumor to the anterior brain vessels [111], [344], [358]. Tumors with supraorbital processes or involvement of the posterior wall of the frontal sinus are not or at least not exclusively treated by endonasal surgery [82].

A macroscopically complete transnasal resection of meningiomas of the anterior cranial fossa (GTR, see above) is successful in 50–90% according to the literature [26], [30], [58], [61], [63], [121], [283], [302], [320], [344], [353], [358], [649], [651].

This success rate is not always better than for transcranial interventions [58], [653]. The same is reported for the results concerning the postoperative vision [651] while other authors confirm an advantage of transnasal surgery [648]. The prognosis of the postoperative vision depends on the size of the tumor [100]; the rate for example of postoperative visual improvement is various and amounts to up to 90% [650]. CSF fistulas occur more often in endoscopically operated cohorts, other surgical complications appear rather in conventionally operated patient cohorts [648], [653].

There are several arguments opposing a too generous indication of endonasal resection of meningiomas of the anterior skull base: alternative, minimally invasive transcranial approaches are at disposition (e.g. supraorbital craniotomy via eyebrow skin incision). The nasal physiology remains intact; the same is true in single cases for small olfactory groove meningiomas regarding olfaction. A transcranial approach allows a broad overview over the entire frontal skullbase and leads to better identification of lateral tumor parts that sometimes cannot be easily delineated in the preoperative imaging. Hidden anatomical areas can be well visualized transcranially with angular optics in the sense of endoscopically assisted techniques.

### 5.4 Craniopharyngiomas

Craniopharyngiomas correspond to squamous cell dysontogenetic tumors of the former craniopharyngeal duct. The tumor can be located in the supra- or intrasellar region or in the 3^rd^ ventricle. Most frequently, a suprasellar location is found with origin in the posterior part of the pituitary stalk dorsal to a ‘prefixed optic chiasm’. In the course of expansion, the tumors show a specific tendency to tissue adhesions at regional neurovascular structures (chiasm, pituitary gland, hypothalamus, thalamus, fornix). In about 50% of the patients, visual field loss is subsequently observed or reduced visual acuity. Frequently even hydrocephalus or endocrinological deficits may occur. It is difficult to predict the exact local growth pattern (extra-arachnoid, subarachnoid, extrapial, partly intraparenchymal-subpial) of a tumor based on imaging. 

The therapeutic concept is based on the resection of the tumor, as far as it is safely possible. Median located tumors can preferably be transnasally approached via a transsphenoid or transplanum-transtuberculum corridor. Advantages are the missing brain retraction, a minimal manipulation and early decompression of the optic system, an early identification of the pituitary gland and the pituitary stalk as well as the protection of delicate vascular structures in the area of the pituitary stalk, the chiasm, and the hypothalamus. If the tumor cannot be resected completely with a justifiable risk, a partial resection or fenestration is performed in case of cystic tumors (in single cases with drainage into the sphenoid sinus) and adjuvant therapy follows (e.g. stereotactic irradiation, intracystic drug instillation) [26], [61], [93], [109], [111], [322], [528], [543], [600], [654].

With regard to the transnasal approach, the relation of the tumor to the infundibulum of the pituitary gland is of special importance. This circumstance is considered in the following classification [168].

- Type 1: preinfundibular tumor.
- Type 2: intrainfundibular tumor. The craniopharyngioma develops within the pituitary stalk.
- Type 3: retroinfundibular tumor. The craniopharyngioma grows from there either in rostral direction into the anterior 3^rd^ ventricle (type 3a) or caudal in direction of the interpeduncular fossa (type 3b).

Other classifications refer to prechiasmatic and postchiasmatic tumors, with or without extension into the 3^rd^ ventricle. Infradiaphragmatic tumors have a comparably better prognosis. They can be classified into sellar and suprasellar tumors [543].

In the literature, data on the completeness of the resection vary. Frequently, in the preoperative analysis a complete resection is not the primary therapeutic objective (see above). If complete removal is planned, it can be realized in about ¾ of the cases – rather with infradiaphragmatic location (80%) of the tumor than with supradiaphragmatic location (66%). The rates are higher compared to transcranial interventions [26], [30], [58], [61], [281], [282], [283], [322], [325], [344], [434], [517], [528]. The major part of tumors of other location is extirpated subtotally; in about 10% of the cases, partial resection is achieved [655]. 

After incomplete resection, clinical recurrence must be expected in 75% of the patients within 2–5 years [92], [654]. Even in cases of complete resection (GTR, see above) recurrences are observed in about 20–40% [26], [109], [131]. Endonasal revision surgeries are generally promising, especially after previous transcranial intervention [445].

Postoperatively, the ophthalmological deficits improve in the majority of the patients (60–80%). This rate is better than for “classical approaches”. The endocrinological results are comparable; in at least 60% of the patients a (sometimes temporary) postoperative diabetes insipidus and in about 50% an endocrinological deficit has to be expected [26], [92], [277], [322], [325], [344], [445], [528], [543].

**Figure 8:**
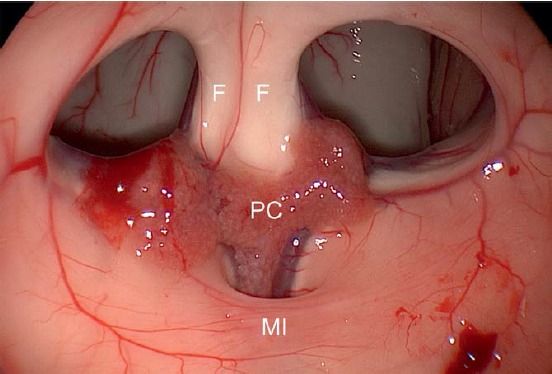
View into the 3^rd^ ventricle with a 45° optic after resection of a craniopharyngioma

Skull base reconstruction after craniopharyngioma surgery is particularly challenging because especially after opening of the 3^rd^ ventricle a high CSF flow must be expected (Figure 8 (Fig. 8)) and the local anatomy does not allow voluminous implantation (see above). Thus the rate of postoperative CSF fistulas is increased [282], [517], [527]. The lethality rate of those interventions, however, is low [543].

### 5.5 Chordomas, chondrosarcomas

Chordomas originate from remnants of the notochord and are located epidurally in the area of the skull, mostly in the midline of the clivus. They are considered as low or middle grade malignomas. Chondrosarcomas originate from primitive mesenchymal cells or from the embryonic cartilaginous matrix of the skull and occur predominantly in the area of the spheno-occipital fissure or at the clivus. Both entities are rare tumors. They are located primarily extradurally, may infiltrate bones, tend to develop recurrences, and do come rapidly in contact with important neurovascular structures. Hence, an oncologically sufficient tumor resection is difficult. The therapy consists of possibly radical tumor resection, often radiotherapy is performed afterwards. In general, a transsphenoid and transclival approach is chosen, in case of need completed by a transpterygoid access [83], [106], [237], [295], [305], [656], [657]. Special reconstructive procedures are only necessary in cases of intracranial-intradural extension of the tumor.

According to the literature, the rate of complete tumor resection (GTR, see above) amounts to 40–80%. This value is higher than for open interventions. If the tumor is larger than 4 cm, the volume is higher than (35–) 80 cm³, or if it shows relevant tumor growth lateral to the paraclival internal carotid artery, a complete transnasal tumor resection is not very likely. In those cases, a subtotal resection of more than 90% of the tumor mass should be achieved [26], [30], [58], [64], [83], [106], [118], [124], [131], [237], [295], [487], [656], [657], [658].

The recurrence rate after first interventions amounts to 26% and after recurrence surgery to 44%. Only one third of the revisions leads to complete tumor resection. The possibility of intraoperative tumor dissemination is discussed; however, it is considered as being relatively improbable in case of transnasal interventions [62], [66], [659]. Residues were observed most frequently below and lateral of the internal carotid artery at the foramen lacerum [26].

### 5.6 Cysts in the area of the sella

In the area of the sella, different cystic lesions occur. Comparably frequent are cystic adenomas and cystic craniopharyngiomas; more rarely, Rathke’s cysts occur as well as epidermoid cysts and arachnoid cysts. The last mentioned ones are only fenestrated and connected to the normal CSF circulation. The wall of cystic adenomas often contains parts of the normal pituitary gland – those parts are protected [281], [284].

Rathke’s cysts develop from Rathke’s pouch and are mostly located in the pars intermedia of the pituitary gland. They are lined by a unilayer ciliary epithelium and occur frequently, but mostly without causing symptoms. Single cysts grow and cause pituitary dysfunction, diabetes insipidus, disturbed vision, and headaches. Sellar cysts are approached via a transsphenoid corridor. The cysts are inspected and opened with an angular optic from caudal behind the anterior lobe of the gland. In case of suprasellar cysts, this step is performed via a transtuberculum-transplanum access. The cyst is drained, as far as possible the wall is resected. According to the literature, complete resection is possible in 60–100% of the cases. The radical detaching of the cystic wall from sensitive structures should be avoided as it threatens small nutritive vessels.

In up to 33%, the cyst refills postoperatively. Clinical recurrences are expected in about 13%. Squamous cell metaplasia of the cystic wall is the most important factor for clinical recurrence. New endocrinological deficits are observed in 4–30% postoperatively [61], [115], [281], [283], [325], [660], [661], [662].

In case of smaller interventions because of Rathke’s cysts, a reconstruction of the sellar floor is not performed [663]. In other cases (e.g. intraoperative CSF flow, excessive thinning of the sellar diaphragm) a typical reconstruction is performed, e.g. with a fat plug. An insertion of tissue in the sella is not recommended by some authors because this measure is associated with a higher recurrence rate [661].

### 5.7 Malignomas

In the context of rhino-neurosurgical malignoma surgery, selected tumors revealing significant involvement of the dura, intracranial growth or in critical neighborhood of important neurovascular structures are treated [52], [664]. In detail those represent very heterogeneous entities and cohorts. Reports are submitted often by specialized centers and cannot be easily transferred to smaller institutions. The individual anatomy and pathology together with the manifold perioperative, organisatory, and finally also ethical impact factors impedes valid investigations of the relative value of endonasal interventions. The most frequently occurring malignomas for endonasal approaches are squamous cell carcinomas, adenocarcinomas, adenoid-cystic carcinomas, esthesio-neuroblastomas, nasopharyngeal carcinomas, and undifferentiated carcinomas (SNUC) [26], [54], [627].

For carefully selected patients presenting with less advanced tumors, results of endonasal surgery seem to be comparable to conventional craniofacial interventions, sometimes even superior. The perioperative morbidity is estimated as being low [54], [91], [96], [299], [302], [397], [627], [665].

In typical cases, at the beginning of the intervention tumor debulking is performed, then subperichondral or subperiostal dissection and resection of the mucosa together with residual tumor parts. Afterwards the dura, if needed including the olfactory bulb on one or both sides is resected [8], [544].

Tumor-free resection margins are much more important for the therapeutic outcome than the type of tumor removal (“en bloc” or piecemeal resection) [26], [75], [96]. Certain entities such as the adenoid-cystic carcinoma usually cannot be completely resected [26], [625]. Positive specimens of the resection margins (R1 resection) in other cases force to further and probably destroying interventions [30]. Recurrences mainly occur locally and indicate a clear deterioration of the overall prognosis [666].

### 5.8 Vascular diseases

If alternative effective approaches are missing and the options of interventional therapy are limited, in single cases also transnasal surgery for intracranial vascular diseases such as aneurysm or AV fistula in or near the midline with ventral orientation can be indicated [26], [309], [667]. There are reports for example about the successful transclival treatment (“clipping”) of aneurysms of the vertebral artery directly at the exit of the PICA or the superior pituitary artery [309], [405].

Based on a comprehensive strategy, in single cases the initial steps of a conventional (pterional) approach were performed first in order to be prepared for intraoperative emergencies [405]. In another case, a large aneurysm of the vertebral artery with compression of the brain stem was treated by a combined approach implying endovascular coiling in combination with transnasal exposition, direct transnasal closure (trapping) and endovascular thrombectomy Such a combination of the modalities seemed to be necessary in order to avoid possible rupture of the aneurysm [668].

Other reports have been published about a transclival approach to treat cavernous malformations in the pons. Inevitably, neurological problems occurred postoperatively that significantly improved after several months [669], [670]. An arterio-venous malformation of the clivus in a 4-year-old child was addressed with transnasal surgery combined with midfacial degloving [671].

## 6 Particularities of the perioperative patient management

### 6.1 Nasal packing, postoperative imaging, postoperative instructions

After pituitary surgeries or transsphenoid standard interventions, nasal packing is not always necessary [18], [306], [324], [386]. In other cases, balloons and various other materials (e.g. gauze, absorbable or non-absorbable sponge tamponades) are applied for nasal packing. Sponge tamponades may be immersed in antibiotic ointment (Bacitracin) or for example soaked gentamicin solution [446].

The materials remain in situ for about 10 days (2–14 days) [2], [383], [105], [163], [295], [317], [446], [491], [519], [537], [543], [554], [561], [594], [622], [672], [673], [674], [675], [676].

Inserted balloons are generally removed after 4–10 days (sometimes even 1 ½ days) [55], [491], [504], [677].

After the intervention, it is important to achieve atraumatic extubation [302]. Following the anesthetic recovery phase, supervision in an intensive care unit is required. Besides the usual parameters, repeatedly neurologic screening tests are performed (e.g. cognitive function, vigilance, motor activity, vision, and field of vision). After interventions at or near the pituitary gland, the serum and urine status are repeatedly evaluated regarding sodium and osmolarity to exclude diabetes insipidus. After controls of the hormone levels, substitution is induced, if needed. Often perioperatively a prophylactic application of corticoids is performed (100 mg hydrocortisone). If necessary, special ophthalmologic controls are initiated [82], [92], [93], [97], [302], [310], [318], [543], [676].

Postoperatively, the head part of the bed is elevated of 30°. If needed, patients keep bed rest for some days and avoid activities for 4–6 weeks that may increase intracranial pressure (Valsalva manoeuver, blowing the nose, pressing, sneezing with closed mouth, lifting of heavy loads). Drinking with straw leads to a negative pressure in the nasopharynx and should be avoided as well. Sometimes laxatives and antihistamines are applied [280], [303], [446], [491], [538], [543], [640], [672], [673], [676], [678]. If an increases CSF pressure is suspected, additionally acetazolamide can be applied [679].

Intensive ENT specific examination and treatment is essential after each rhino-neurosurgical intervention. It is recommended for mucosal care, prophylaxis and identification of healing disturbances or complications [278], [304], [310], [676].

#### 6.1.1 Postoperative imaging

On the 1^st^ or 2^nd^ postoperative day, imaging (CT scan or MRI, more rarely both modalities) is performed. The aim is the control of the surgical site (exclusion of residual tumor and also secondary local damage) with its special defect closure (position of transplants or implants); disturbing wound reactions at that time are not yet obvious. After interventions of longer duration with prolonged anesthetic recovery phase, CT scan is often induced immediately, followed by MRI after 48 hours. Further control of the findings by means of CT scan or MRI is performed at least 3 months after the intervention [82], [92], [93], [163], [302], [317], [325], [383], [519], [531], [674], [676], [678].

### 6.2 Lumbar drainage

The indication for application of lumbar drainage is described in many different ways. Some authors promote explicitly a broad spectrum of indications [94]. In case of restrictive indication, the forced immobilization of the patient and the special complications of drainage are mentioned. It is well-known that e.g. an accidentally or wrongly performed high drainage volume may be associated with pneumocephalus, herniation of brain tissue, or displacement of implantations at the skull base. Other complications may be a disconnection of the drainage system with the risk of ascending infection (wound infection, meningitis), persisting lumbar CSF flow after removal of the drainage and dislocation and retention of the catheter tip when removing the system. In single cases, a temporary paresis of the abducens nerve may occur because of the lumbar drainage, most likely as consequence of the suction effect. The cumulative complication rate of lumbar drainage amounts partly to 13% [277]. Generally, problems with the management of drainage seem to occur more frequently in patients who are changing medical departments in the course of their treatment [82], [286], [380], [680], [681].

Drainages are useful if an increased intracranial pressure is measured, i.e. in association to significantly increased body weight or hints of hydrocephalus in the imaging or spontaneously appearing CSF fistulas. Furthermore, drainage is indicated in intraoperative high-volume fistulas such as for example after transtuberculum interventions or after opening of the ventricle as well as after radio-chemo-therapy. In those cases, the risk of postoperative CSF leak is increased [36], [55], [61], [93], [97], [98], [277], [286], [295], [312], [516], [561], [681]. Similarly, general lumbar drainage is recommended by some authors in revision surgeries or in cases of large dura defects of more than 3 cm [383], [516]. Other authors limit such recommendations for example to defects at the clivus of more than 2 cm in size [66] or leave the decision up to the surgeon with an individual estimation of the risks [61], [676].

According to alternative recommendations in literature, lumbar drainage is not performed in all cases or explicitly in certain disease entities [102], [320], [446], [530], [538], [543], [674], [675], partly mentioning a high risk of secondary CSF fistulas after lumbar drainage [611].

Lumbar drainage is generally applied for 1–7 days. The flow rate is normally 5–10 ml per hour [2], [82], [92], [94], [97], [118], [302], [310], [380], [488], [504], [508], [520], [546]. An oral application of acetazolamide (2×500 mg) reduces the CSF pressure in positive pressure patients; it is useful as additional part of therapy in such patient populations [679].

In single cases it is recommended to induce saline solution at the end of surgery as therapy of pneumocephalus (30–50 cm³ for 10–15 min) via the lumbar drainage. Patients with such substitutional therapy should have less complaints and a shorter hospitalization [673].

### 6.3 Perioperative antibiotic therapy

For traditional craniotomies, a benefit of perioperative antibiosis is described [682]. In cases of rhino-neurosurgical interventions, there is no consensus in literature regarding this aspect. The risk of infection is generally considered as being low in interventions revealing a regular course [681].

The indication of antibiotic therapy depends on accompanying factors. After pituitary adenoma surgery, it is applied for example only in cases where intra- or postoperative CSF flow is observed [304]. Other authors indicate antibiosis only after nasoseptal flap application [677].

Concerning systemic therapy, preferably cephalosporins are considered [41], [82], [183], [295], [531], [673]. Alternative drugs are ampicillin-sulbactam, clarithromycin, vancomycin, aminoglycosides, or metronidazole, partly as single treatment or in combination [18], [102], [310], [676]. The application occurs orally or i.v. [102]. Gentamicin is sometimes applied as nasal spray for 14 days postoperatively [683].

The duration of antibiotic therapy amounts to 1–10 postoperative days [163], [446], [676], [683]. In doubtful cases, the majority of authors promotes therapy over the duration of inserted nasal packing or over the time of lumbar drainage [2], [55], [94], [303], [320], [446], [513], [514], [520], [531], [543], [674], [676].

### 6.4 Multimodal therapy

If tumors affect critical neurovascular structures or develop in unfavorable locations, it is often not possible to perform complete resection. In cases of very advanced malignomas, a primary chemo-radiotherapy may be discussed – surgery would then be performed as secondary salvage surgery, e.g. for residual tumor parts [105].

In other cases, adjuvant radiotherapy is performed about 6–8 weeks after surgery. Defect covering with pedicled flaps does not oppose such a schedule [566], [666], [684]. According to the literature such tumor entities might be: meningiomas, pituitary adenomas, glomus tumors, cavernous hemangiomas, craniopharyngiomas, chondromas, chordomas, squamous cell carcinomas, adeno-carcinomas, chondrosarcomas, adenoid cystic carcinomas, esthesio-neuroblastoma, nasopharyngeal carcinomas, and melanomas. Different types of irradiation are applied (stereotactic radiosurgery / stereotactic hypofractionated vs. conventional fractionated irradiation, proton radiation, rarely also brachytherapy) – the different types of irradiation, however, have not been investigated comparatively regarding adjuvant radiotherapy of skull base disease. Stereotactic radiosurgery is expected to achieve higher response rates in comparison to conventional radiation. Clivus chordomas are preferably irradiated with protons. The maximal dose in single patient populations amounted to 18–61 Gy [101], [109], [124], [132], [384], [398], [455], [493], [528], [529], [626], [666], [685], [686], [687].

In case of systemic disease or advanced extension of the tumor into the mucosa, also adjuvant or neoadjuvant chemotherapy or chemo-radiotherapy is performed [54], [142], [276], [317], [322], [414], [688]. According protocols were also developed for children [275].

High-dose radiotherapy leads to side effects that express among other aspects as disturbed vision or endocrine dysfunction [65]. Possible headaches, seizures, and cognitive impairment often regress after several months [686]. 10% of the patients have to expect relevant neurological side effects after irradiation of the clivus [192]. Many patients complain about nasal secretion with crusting for about one year – the term of “prolonged crusting” is used after more than 6 months [108], [684], [689]. In single cases, radionecroses of the frontal brain or the pituitary gland or postradiogenic blindness were observed as irradiation damages [107], [689]. Another report describes osteoradionecrosis of the clivus with a 3 months delayed lesion of the internal carotid artery [142].

Nonetheless, the combination of surgery and stereotactic radiation offers the most effective therapeutic benefit for the mentioned entities [300]. It is proven that surgery with postoperative radiation achieves the best outcome for esthesio-neuroblastoma [105]. In contrast, the local control of melanomas is improved, however, the overall survival did not change [54]. Postoperative radiotherapy cannot avoid later progression rates of 29% in recurrent craniopharyngiomas [654].

## 7 Complications, postoperative care

Part of the complications of rhino-neurosurgical interventions corresponds to undesired events as they generally may occur also in routine surgery of the paranasal sinuses [399].

Basically, the type and rate of complications depend also on the type, location, extension of the lesion, the type and performance of surgery as well as general or specific patient-related factors.

### 7.1 Complications

Patients undergoing rhino-neurosurgical interventions are subjected to the risk of long-lasting surgeries. In about 3% of the cases, general systemic complications have to be expected (pneumonia, leg vein thrombosis, pulmonary embolism, multiorgan dysfunction, asystole). Especially older patients are affected (>60 years) [68], [183].

Furthermore, a series of well-known rhinosurgical complications may occur associated with the creation of the special surgical corridors. Examples are postoperative bleedings, synechia, sinusitis, mucoceles, atrophic rhinitis, bleeding into the orbita, injury of the medial rectus muscle, or pneumocephalus and tension pneumocephalus [277], [399]. After rhino-neurosurgical interventions, those known complications, however, are associated with specific requirements. In cases of postoperative bleedings for example, the possibility of damage of greater vessels or the risk of intracranial extension of the bleeding must be taken into consideration. Postoperative sinusitis should be confirmed early by means of swab and treated immediately in order to avoid ascending infection [277].

Particularly tragic is a case report about a misplaced gastric tube after endoscopic intervention with insertion of the tube through the reconstructed clivus into the spinal canal [690].

In the following, other special complications are described. They may occur acutely or with clear delay [68], [691].

Regarding the incidence of complications, the literature presents very different data – the variation corresponds to the high variability of findings, interventions, surgical techniques, and expertise. In large series of specialized centers, a complication rate of rhino-neurosurgical interventions appears to be comparable to conventional surgeries [68].

Generally, roughly simplifying the situation, special complications of rhino-neurosurgical interventions must be expected in up to 10%: small case series even report of 20% (–50%) [26], [96], [277], [479], [543]. In other references, severe complications are mentioned in 0.1–6% of the cases [96], [310]. The direct lethality is very low according to the literature, in single series it amounts to <1% (0.24–3%) [68], [100], [633].

#### 7.1.1 Postoperative CSF fistulas 

Metaanalyses on complication rates of endoscopic interventions at the pituitary gland reveal a rate of postoperative CSF fistulas of 2% [526], [633]. In historical collectives after extended rhino-neurosurgical interventions the rate amounted initially to inacceptable 40%. However, the gain of surgical expertise and the application of pedicled local mucosal flaps could reduce this rate to 5% under favorable circumstances (data of the literature: 0–40%) [26], [36], [55], [61], [64], [68], [92], [94], [100], [117], [118], [131], [183], [277], [283], [290], [302], [322], [344], [355], [384], [446], [504], [517], [543], [546], [599], [600], [649], [651], [674], [692], [693]. This amount should no longer be generally different from the one of craniofacial interventions [504]. The fistulas mostly occur directly after surgery, more rarely in a timely interval [691]. The following factors indicate an increased individual risk of postoperative CSF fistulas: higher complexity of the intervention, intraoperative opening of cisterns or ventricles, condition after previous surgeries or irradiation, advanced age or high BMI of the patient, existing Cushing’s disease. Pinpointing the reliability of pedicled flaps, there is a controverse discussion if the risk of CSF fistula always increases with the size of the dura defect [68], [504], [611], [674], [692], [693]. The same is true for the location of the defects: some authors consider the anterior skull base and the clivus as particularly sensitive [504], [611]. Other authors explicitly deny the specific risk of both locations and attribute an increased risk to transplanum-transtuberculum interventions [277], [446], [508], [546]. The dura opening in context of those interventions has a comparably difficult geometry. Insertion of transplants (inlay) does not guarantee a natural counterpressure by the frontal brain – moreover, inlay transplantations are problematic due to mass effects caused by swelling with pressure on the optic tract and possible intradural migration of the material with disturbed CSF pathways [277], [446].

Meningiomas, craniopharyngiomas, and chordomas have relatively higher postoperative rates of fistula development, other authors however deny a general influence of pathological entities on the success of defect closure [26], [380], [515], [546]. Similar holds true for the “first 10 cases” and duration of surgery of more than 6 hours [611].

Together with CSF fistulas, often pneumocephalus, in single cases also tension pneumocephalus, or even intraventricular tension pneumocephalus are observed [384], [446], [688], [694]. In one case, an intraventricular tension pneumocephalus required emergency revisional surgery with ventricular drainage one week after the intervention [695]. In another case, an extensive loss of CSF caused a subdural hematoma [696]. In the absence of a fistula, also a secondary (meningo-) encephalocele may develop over a duration of months or years [107], [120].

If a CSF fistula occurs postoperatively, the correct position of the transplants is examined by imaging first and a relevant pneumatocephalus is excluded. If no lumbar drainage is placed, it may be justified as first measure according to the individual needs. If drainage is already placed, revision surgery may immediately be indicated [492], [508].

#### 7.1.2 Infectious complications

Meningitis may appear directly postoperatively or with a clear timely latency after surgery [697]. Generally every postoperative meningitis can be life-threatening [698].

According to the review of the literature, the risk after endoscopic endonasal skull base interventions amounts to <2% (range: 0–14%) [26], [36], [55], [60], [61], [92], [99], [117], [283], [355], [508], [693], [699], [700]. The rate is not increased in comparison to patient cohorts undergoing conventional interventions [693]. The favorable rate is mainly due to perioperative antibiotic prophylaxis and frequent intraoperative rinsing [105]. However, the rate increases to about 13% in patients with postoperative CSF fistula [700]. Further risk factors are: male gender, previous surgeries, pre-existing CSF drainage, extensive interventions of durations of more than 4 hours, meningiomas with broad base on the dura [360], [508], [693]. In single case series, a higher frequency of meningitis reflects an increased willingness of the surgeons to take risks [380], [515].

If meningitis occurs without apparent CSF flow, always the possible infection of lumbar drainage must be considered, if present [701].

After transsphenoid surgeries, the rate of postoperative sinusitis of the sphenoid sinus amounts to 1–30%. Certain transplantation materials inserted at the sella (e.g. septal bone) are particularly sensitive for dislocations with subsequent sinusitis caused by staphylococcus or pseudomonas [526], [635].

Beside meningitis, also frontal lobe abscesses may occur [689]. In case of intracranial infectious foci, staphylococcus aureus, gram-negative germs, or poly-microbial infection must be expected [277].

Meningitis is treated with antibiotics [283]. All infected alloplastic materials have to be removed [277].

#### 7.1.3 Endocrine complications

Generally, after interventions at or near the pituitary gland and the pituitary stalk a newly occurring or increasing pituitary insufficiency can be expected [18], [131], [277], [280], [694]. The rate for routinely performed pituitary gland interventions amounts to about 3% [60].

In up to 15%, temporary diabetes insipidus is observed. In about 1–2%, the posterior pituitary lobe permanently loses its function [427], [633].

A special type of postoperative pituitary dysfunction is the syndrome of inadequate ADH secretion (SIADH – syndrome of inappropriate antidiuretic hormone secretion). It occurs in about 8% of the patients 3-10 days after the intervention. The respective postoperative electrolytic imbalances can be life-threatening. An exact monitoring of the patient with control of the laboratory parameters and an early reaction are essential [60], [277], [543].

Certain patients suffering from acromegaly require perioperatively additional attention regarding e.g. obstructive sleep apnea (OSAS) [194].

In cases of complex rhino-neurosurgical interventions, the risk of endocrine complications is individually further increased. Temporary and also permanent postoperative hormone deficits often occur for example in craniopharyngioma patients [61]. The rate of diabetes insipidus increases in single series to up to 66% or 79% based on literature reviews [282], [649].

#### 7.1.4 Neurological complications

According to the literature, various neurological deficits must be expected postoperatively in about 5–40% of the cases [66], [97], [106], [117], [124], [282], [355], [651].

For example, the optic nerve, the abducens nerve, or the oculomotor nerve in the direct surgical site may be damaged mechanically [18], [131], [165], [277], [448], [694]. Postoperative problems after resection or injury of the Vidian nerve are often not reported (see below) [624].

Rarely, herniation of parts of the optic tract and the 3^rd^ ventricle caused by operatively opened sella was observed [663]. The local pressure may also cause intrasellar hematoma leading to reduced vision [507]. The risk of pressure-induced damage caused by extensive insertion of tissue in the context of dura reconstruction was already mentioned [60], [115]. The same is true for hydrocephalus after excessive subdural insertion of fatty tissue or oxidized cellulose with fibrin glue [657].

The resection of suprasellar meningiomas leads to postoperatively reduced vision in 0–40% [68], [97], [699]. In contrast, it must be mentioned that a postoperative improvement of visual disturbances can be achieved in 30–80% after transnasal interventions [651], [699].

In single cases, bilateral blindness, hemi- or monopareses, bleeding or infarction of the brain stem (partly with lethal outcome), cognitive dysfunctions, and postoperative seizures were observed [18], [60], [68], [92], [98], [106], [277], [528], [655], [657], [702]. Single cases of hemiplegia or ataxia occurred postoperatively with relevant delay [87]. After damage of the hypothalamus, hyperphagia may develop [92], [543].

#### 7.1.5 Vascular complications

In about 0.7–5% after rhino-neurosurgical intervention a relevant, up to 10 days delayed postoperative bleeding in form of epistaxis must be expected. In general, the branches of the sphenopalatine artery are the source of the bleeding. In some minor cases, also tumor residues can be the origin [60], [98], [194], [507], [703].

In case of extensive interventions, regularly the need of blood transfusions may arise. The transfusion rate amounts to about 25% in single cohorts [689].

In up to 10% of the cases, hematomas in the area of surgery are described, e.g. with intra- or suprasellar location. Subdural hematomas were observed in 5–17% of the patients according to single reports [18], [98], [528]. They may become apparent immediately or as late subdural hematomas with a timely latency of 3 weeks to 4 months for example based on headaches, neurological deficits or seizures [107], [322], [381], [655], [678]. Events of epidural hematoma caused by the pin of the sharp fixation of the head in a child [696] or a subdural hematoma after insertion of lumbar drainage [704] are rare.

In rare single cases, immediately after surgery or with delay a life-threatening, often lethal intracranial or intraventricular bleeding occurred [18], [120], [304], [353], [384].

Generally direct vascular damage is reported in about 0.9–5% of the surgeries. This concerns smaller vessels with and without neurological subsequent damage (e.g. subchiasmatic vessels) or greater vessels (anterior cerebral artery) with immediate neurological deficit [68], [282], [651]. In many cases, the internal carotid artery is exposed in transsellar, transclival, or transpterygoid interventions, accompanied by the risk of primary injury or secondary bleeding [131], [203], [289], [448]. Direct damages are mentioned with an incidence of 0.2–1.8% in single reports. The majority of the lesions occurs on the left side [177], [194], [203], [703]. A very rare event is the rupture of the vessel in the parapharyngeal space [276].

Smaller lesions of the carotid artery by tearing off a small arterial branch can be treated with bipolar coagulation or by covering the defect with hemostyptic materials. Larger injuries primarily require local tamponades, if necessary clamping the vessel with clips. A high blood loss (data in the literature: 400–4200 ml) in the context of primary treatment can be expected. Afterwards, emergency neuroradiological control is performed, probably applying an endovascular stent or a definitive closure of the vessel (coiling). About half of the arteries can be preserved – mostly after small lesions [18], [36], [106], [203], [290], [656], [694]. A continued postoperative care with repeated angiography (preferably CT-Angiography) is required – even several months or years after such trauma, rupture of a developing pseudoaneurysm may occur [62], [203], [696], [705]. This risk of delayed bleeding from traumatic aneurysm also involves Willis’ circle or frontopolar arteries [183], [277].

#### 7.1.6 ENT specific postoperative effects and complications, hyposmia

Rhino-neurosurgical interventions are a special challenge to rhinological expertise:

The sinonasal morbidity after transnasal interventions is always increased over a longer time period. Subjectively disturbing crusting and viscous secretion can nearly always be expected for at least 3 (–9) months [2], [83], [507], [544], [614], [666], [676], [706], [707], [708], [709]. Negative factors are the application of nasoseptal or other mucosal flaps, excessive resection of turbinate tissue, big surgical cavities, xenogenic transplantations, and radiotherapy. The donor defect of nasoseptal flaps requires intensive local care until re-epithelization is finished after about 90 days. The time may be shorter after raising other flaps, e.g. from the inferior turbinates. Generally the nasal physiology is disturbed postoperatively for a longer time also without application of flaps – chronic crusting is the term used in literature after more than 6 months of complaints [91], [94], [491], [504], [544], [624], [672], [706], [709].

Postoperatively, synechia may occur frequently [432], [433], [624]. Rarely, empty nose syndrome is observed as maximum variation of chronic rhinitis sicca [267], [710].

Postoperatively, persisting mucosal congestion may appear in the remaining paranasal sinuses (especially in the anterior ethmoid sinus and in the maxillary sinus after resection of the medial concha and generous antrostomy) [711]. Postoperative sinusitis can develop directly or after some time [18], [131], [280], [304], [688].

By use of endoscopes, high-speed drills, or ultrasound aspirators, sometimes temperatures in the surgical field of up to 45°C (top values of 62°C) may occur. Those temperatures can damage the particularly delicate neural neighboring structures; thermal damages are also sometimes observed at the nasal entrance [712].

Postoperative hyposmia or anosmia may be an inevitable consequence of necessary resection at the skull base. Apart from this functional loss, the rate of postoperative disturbances of olfaction increases parallel to the extent of performed intranasal tissue resections [277], [432].

Beside a resection of parts of the middle and superior turbinates, posterior septectomy and especially the application of nasoseptal flaps may contribute to postoperative functional disturbances of the olfactory mucosa [277], [708]. In single reports, only few patients are concerned (9%) [713]. Other authors observed only a temporary effect, that exists in up to 50% of the patients for example after routinely performed pituitary surgeries for about 3–6 months [591], [621], [708], [714]. The reason for this seems to be a secondary edema development in the surgical site [714]. Other authors indicate a significant impairment of the olfaction and increased headaches even 6 months after application of nasoseptal flaps [622], [715], [716]. As modifications of the incision of the flaps cannot completely exclude those functional losses, a dorsal septal defect with consecutively altered air passage may also play a causative role (conductive olfaction loss) [716]. In total, the quality of life improved in the postoperative interval [715].

After extensive tissue mobilizations and resections inside the midface, for example after transpterygoid interventions, further postoperative functional disturbances can be expected. Beside local pain, those may be infraorbital hypestheia (V_2_), Horner’s syndrome, sensitivity disorders at the palate or teeth, Eustachian tube malfunction with serous otitis media, dysgeusia, or halitosis [101], [116], [267], [276], [455], [596], [624].

Generally, the postoperative morbidity after transpterygoid nasopharyngectomy is considerable [142]. In some cases, transodontoid interventions may lead to velo-pharyngeal incompetence [104]. After detaching the pterygoid muscles or after removal or destabilization of the pterygoid process, temporary trismus or general chewing disorders are complained [71], [258], [455]. If bigger parts of the anterior medial wall of the maxillary sinus have to be removed for an advanced endonasal approach according to Denker, an exterior deformation of the lateral nasal base (loss of lateral alar support) may result [276]. Generally, however, postoperative growth disorders of the midface do not occur in children [131].

A lesion of the pterygopalatine ganglion might lead to hypolacrimation with conjunctivitis sicca or keratitis [26], [116], [254], [267], [276]. However, it is denied that the mere resection of the Vidian nerve causes relevant problems in this context (see above) [250], [407].

### 7.2 Hospitalization, special care after hospitalization

#### 7.2.1 Hospitalization

The extreme heterogeneity of the patient populations is also reflected in the very different data of the literature regarding hospitalization.

Patients having undergone interventions for pituitary adenomas are hospitalized for an average of 3–5 days postoperatively [427], [377], [390], [717]. In other patient cohorts, 80% of the cases are discharged already after 24 hours [331], [324].

A similar hospitalization of 2–5 days is also mentioned for other rhino-neurosurgical interventions (e.g. craniopharyngiomas, esthesio-neuroblastoma, cholesterol cysts of the petrous apex, interventions at the clivus) [61], [82], [96], [97], [288], [302], [381], [455], [543], [630], [672], [683], [687]. In those cases, the patients are recalled for removal of residual packing material about 7–10 days postoperatively [303]. After skull base interventions, some patients are supervised only for one night in a regular ward [2]. Other authors recommend general hospitalization of at least 5 days in order to early identify possibly developing meningitis [90].

For other patient populations, clearly longer hospitalization times of up to 44 days are described (average 6, 7, 8, or 9 days) [5], [63], [275], [281], [315], [353], [384], [465], [626], [699]. The duration of hospitalization increases naturally if postoperative complications such as a CSF fistula or diabetes insipidus occur [318].

#### 7.2.2 Special care after hospitalization

After interventions for malignomas, in the first year control endoscopies should be performed every month, MRI every 4 months. In the second year, endoscopy is suitable every two months, additional MRI every 6 months. Later both modalities can be planned every 6 months [9], [108], [275]. Suspicious mucosal foci are examined by biopsy, additional ultrasound examinations of the neck are performed (e.g. for esthesio-neuroblastoma every 6 months for 2 years) and x-ray of the thorax once a year [529], if necessary, also PET [2]. The patients are introduced in specific tumor consultations with life-long follow-up [2], [666].

According to the situation, in case of benign tumors, modified follow-up schemes are described, e.g. routinely performed endocrine controls (after pituitary gland interventions 3 weeks to 3 months postoperatively, then 6 months postoperatively, later in annual intervals). If pre- or perioperatively problems occurred, a repeated ophthalmologic examinations is performed. Nearly regularly MRI after about 3 months (in single cases again after 6 and 12 months) is recommended. Imaging is continued for a longer time in annual intervals [18], [59], [92], [280], [283], [325], [543].

After all transnasal interventions, outpatient endoscopic care should be performed by ENT specialists in regular intervals [59], [92], [379], [677]. For example, protocols are described with examinations on the 10^th^ postoperative day as well as after 6, 12, and 24 weeks [546].

In single cases, special requirements have to be observed after extensive interventions, which was already mentioned: e.g. large cavities or a septal defect at the nasal septum with nasoseptal flap covering require postoperative care once or twice a week over a time of at least 2–3 months [68], [562], [622]. Generally, several daily rinsing procedures (‘nasal doushing’) are recommended for at least 6 months [594]. The nasal care is supported in some regimens by application of macrolids for 2–3 months or by adding antibiotics to the rinsing solution [432], [533]. For prophylaxis of postoperative chewing disorders after transpterygoid interventions, special physiotherapy with prescription of training devices (stretching devices) is recommended [455].

## Conclusions

Internationally, rhino-neurosurgery has been established in many centers as an interdisciplinary field. For the constituting surgery teams and for patients special new aspects must be observed.

Particular requirements of the ENT surgeon are:

- The interventions require a specific technique of tissue handling, especially in the area of anatomic structures near the brain (internal debulking with subsequent mobilization of the tumor capsula; avoiding of traction of the tissue; protection of very small vessels) [55], [718].
- The completeness of tumor resection with adequate tumor-free resection margins ranks different with the background of the individual risk profile of the preparation.
- In the context of teamwork (sometimes 3 or 4 hands) the responsibility of the ENT surgeon is mainly limited to the approach and the reconstruction.
- Patients require mostly intensive postoperative ENT specific care.
- In many cases, utilization of ENT resources is not optimally supported with regard to medical economy.

Special requirements of the neurosurgeons are:

- In comparison to microscopy, endoscopes have a different, but mostly better view. If necessary, angled optics allow a better range of instrumental action. The missing 3D effect is compensated by dynamic endoscope control (HDTV).
- Teamwork (3 or 4 hands) required interdisciplinary consultation and routines.
- Protective preparation techniques in the area of the ENT specific corridors may have a significant influence on postoperative morbidity (preservation of nasal and olfactory mucosa) and should be respected.

Special aspects of interdisciplinary cooperation:

- Both disciplines should be present during the whole intervention. The significant surgical steps (intradural preparation) are performed by means of 3 or 4 hands technique.
- Durations of surgery are long.
- There is a special longer learning curve, individually and in the teams. This leads to new questions with regard to education and training.

Special aspects of the patient:

- Rhino-neurosurgical interventions lead to no or only minimally esthetic deficits (no visible skin incisions, no osteotomies).
- In children no relevant irritations of growth centers in the face occur.
- Surgical manipulations are comparable protective (mostly direct approach to the lesion with early relief of damaged surrounding structures or pressure on those structures, no brain retraction, comparatively few manipulation at neurovascular structures, inspection and control of hidden anatomical areas by means of optics with an angled view).
- The interventions are comparably protective (short hospitalization in intensive care units, low blood loss, reduced hospitalization, reduced general recovery).
- Postoperatively, longer lasting rhinological morbidity must be expected [12], [55], [305], [307], [323], [665].

The interdisciplinary rhino-neurosurgery is a challenging field of activity that is continuously developing. On the one hand this regards the technical aspect. The general introduction of 3D endoscopy will probably lead to further improvements, solution of still existing insufficiencies provided (e.g. bulky endoscopes, limited field of view, impaired image definition and contrast; likeliness of contamination) [26], [56], [111], [113], [290], [719], [720], [721], [722], [723], [724], [725]. Other developments are expected in the intraoperative imaging or generally image processing and fusion with input of virtual data [10], [726], [321].

Additionally, navigation systems are continuously optimized, e.g. with implementation of feedback options [727]. In the future, computer-assisted calculations will allow to submit these interventions to multiportal surgery, perhaps in combination with miniaturized traditional accesses (e.g. supraorbital mini-craniotomy, transorbital corridor) [64], [78], [85], [89], [728], [729], [730], [731]. In this context, also robotic surgery will have an influence which is currently limited due to narrow surgical corridors in the nose, the sharp angles of inserted instruments, missing tactile feedback, and finally economic reasons [10], [26], [290], [375], [732], [733], [734], [735], [736], [737], [738].

Even conventional instruments are further optimized, so for example by refinements revealing flexible tips, control devices, or the development of special dissectors. The same applies for endoscopes, e.g. as further miniaturization, chip on the tip technique, with variable angle of view or in combination with innovative endoscope holders. Currently tested are technical systems to treat an injured internal carotid artery [12], [26], [330], [719], [739], [740], [741], [742], [743].

The progressing development leads to a steady increase of efforts, costs, and requirements with regard to the quality management. Reports in the literature about learning curves with relation to relevant parameters (completeness of tumor resection, severe side effects) [124], [358] allow a discussion of quality indicators. From some sides, the accreditation of special rhino-neurosurgical centers is recommended as being necessary [744], others claim the development of the special field of endoneurosurgery [354] or a subspecialization as skull base surgeon [1].

## Notes

### Acknowledgements

The authors thank Mrs. Constanze Erdmann (Greifswald) and Mrs. Madlen Hackbart (Greifswald) for their valuable and highly estimated efforts regarding the procurement of the essential literature.

The authors thank Mrs. Susanne Zapf (Marburg) for translation of the German manuscript.

### Competing interests

The authors disclose the following competing interests: consulting activities for the companies Storz and Medtronic.

## Figures and Tables

**Table 1:**
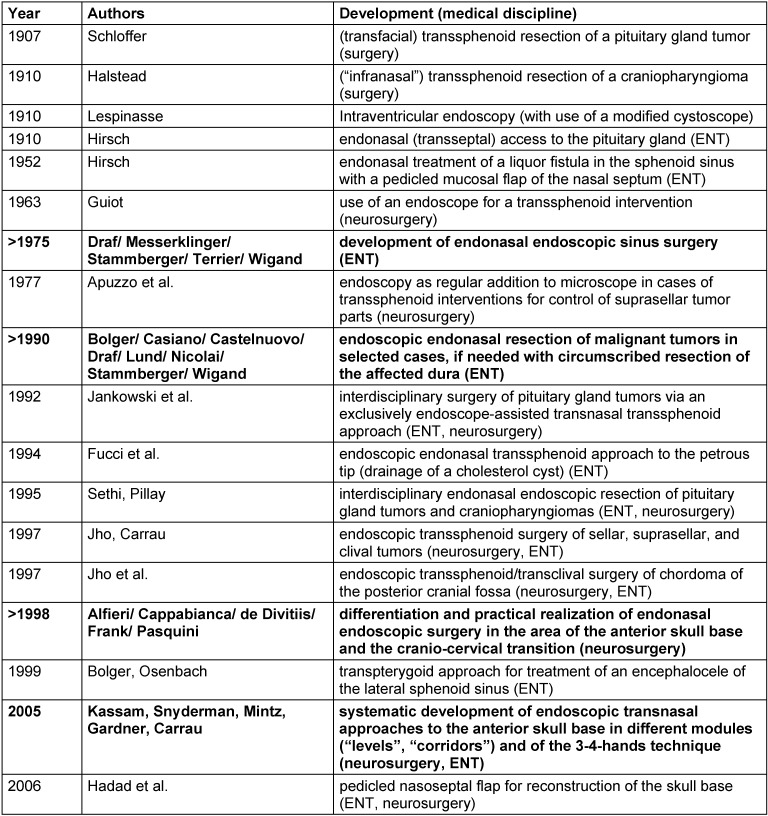
Timetable – interdisciplinary milestones regarding the development of endoscopic rhino-neurosurgical interventions [3, 6-9, 81, 298, 316, 331, 349, 383, 393, 427, 548, 552, 684, 726, 745-51]

**Table 2:**
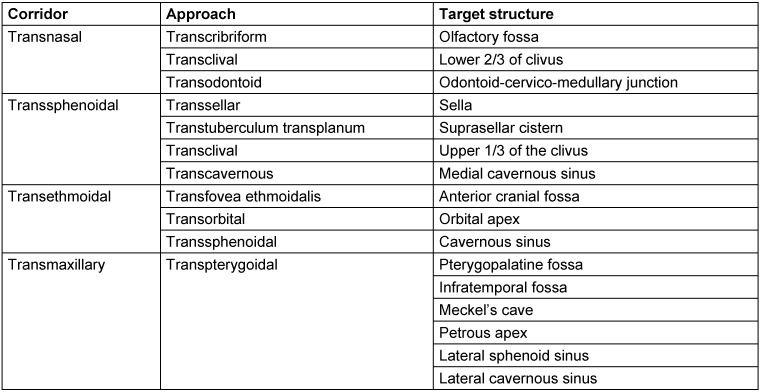
Corridors, approaches, and target structures of rhino-neurosurgical interventions according to Schwartz et al. [30, 67]

**Table 3:**
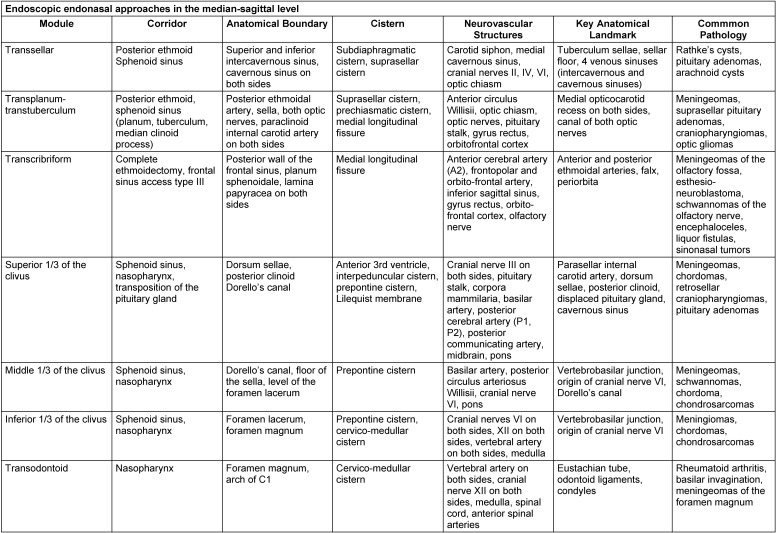
Classification of endoscopic endonasal skull base procedures, accesses of the median-sagittal level: modules and corridors with their parameters and the most frequent basic pathological processes according to Kassam et al. [55, 68]

**Table 4:**
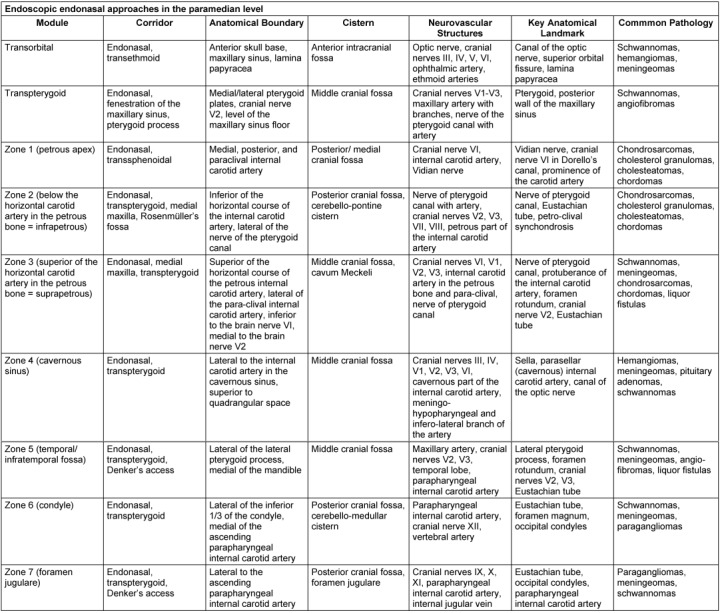
Classification of endoscopic endonasal skull base procedures, accesses of the paramedian level: modules and corridors with their parameters and the most frequent basic pathological processes according to Kassam et al. [68] (nerve of the pterygoid canal = Vidian nerve)

**Table 5:**
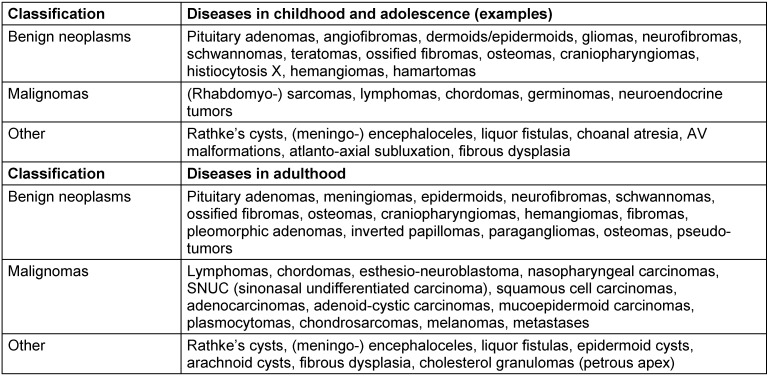
Often used indication (examples) for endonasal endoscopic skull base surgery according to the current literature [extended according to 30, 41, 72, 131, 279, 752]

**Table 6:**
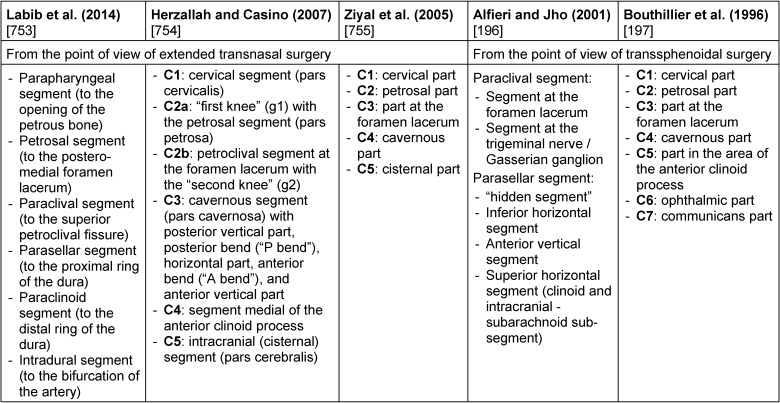
Parts of the course of the internal carotid artery

**Table 7:**
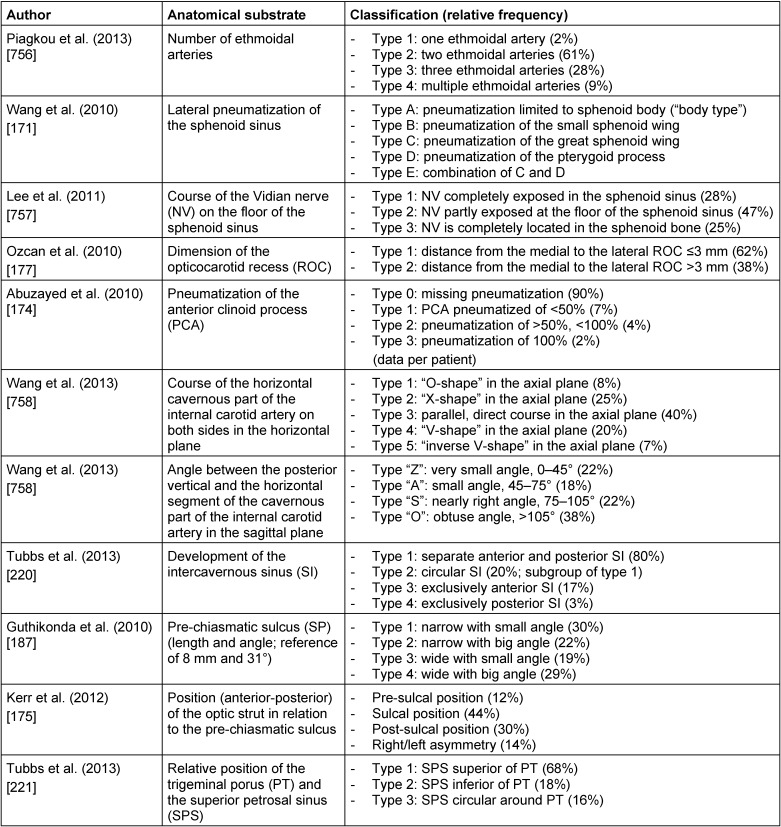
Examples of current anatomical classification systems relevant for the field of rhino-neurosurgery

**Table 8:**
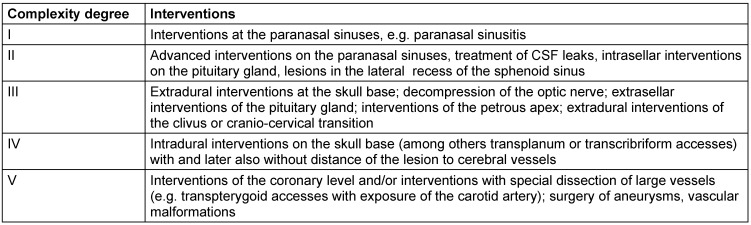
Training program with gradually increased difficulty levels of endonasal rhinological and rhino-neurosurgical interventions (simplified and summarized based on [36, 41, 55, 68, 354, 376])

**Table 9:**
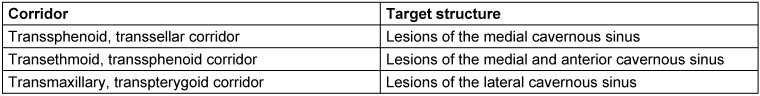
Endoscopic transnasal access to the cavernous sinus [449]

**Table 10:**
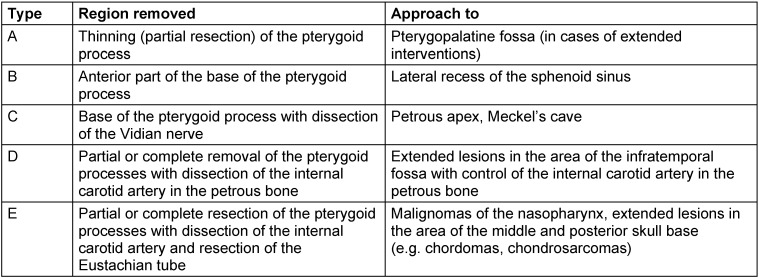
Subtypes of the transpterygoid approach according to Kasemsiri et al. [71]

**Table 11:**
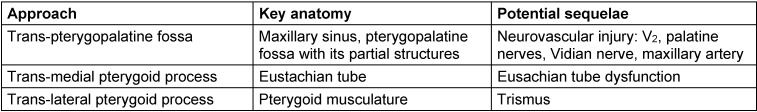
Approaches to the pterygopalatine fossa, the infratemporal fossa, or the parapharyngeal space according to Taylor et al. [455]

**Table 12:**
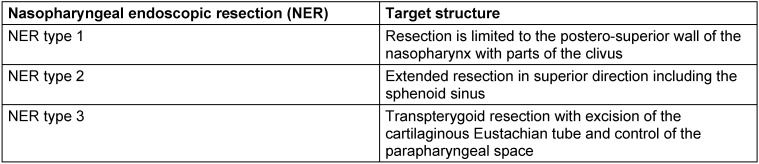
Subtypes of nasopharyngeal endoscopic resection (NER) according to Castelnuovo et al. [126, 143]
